# Advances in the development and optimization strategies of the hemostatic biomaterials

**DOI:** 10.3389/fbioe.2022.1062676

**Published:** 2023-01-11

**Authors:** Yayuan Guo, Nanqiong Cheng, Hongxiao Sun, Jianing Hou, Yuchen Zhang, Du Wang, Wei Zhang, Zhuoyue Chen

**Affiliations:** ^1^ Faculty of Life Science, Northwest University, Xi’an, Shaanxi Province, China; ^2^ School of Medicine, Northwest University, Xi’an, Shaanxi Province, China

**Keywords:** coagulation mechanism, hemostatic biomaterials, modified hemostatic biomaterials, hemostatic strategies, clinic application

## Abstract

Most injuries are accompanied by acute bleeding. Hemostasis is necessary to relieve pain and reduce mortality in these accidents. In recent years, the traditional hemostatic materials, including inorganic, protein-based, polysaccharide-based and synthetic materials have been widely used in the clinic. The most prominent of these are biodegradable collagen sponges (Helistat^®^, United States), gelatin sponges (Ethicon^®^, SURGIFOAM^®^, United States), chitosan (AllaQuixTM, ChitoSAMTM, United States), cellulose (Tabotamp^®^, SURGICEL^®^, United States), and the newly investigated extracellular matrix gels, etc. Although these materials have excellent hemostatic properties, they also have their advantages and disadvantages. In this review, the performance characteristics, hemostatic effects, applications and hemostatic mechanisms of various biomaterials mentioned above are presented, followed by several strategies to improve hemostasis, including modification of single materials, blending of multiple materials, design of self-assembled peptides and their hybrid materials. Finally, the exploration of more novel hemostatic biomaterials and relative coagulation mechanisms will be essential for future research on hemostatic methods.

## Introduction

Uncontrollable bleeding caused by war, accident, trauma, and surgery procedures, is a major problem while performing surgery. Without timely and effective hemostasis, patients often have the risk of causing complications and even life-threatening situations. For instance, 50% of the deaths in the military were caused by excessive bleeding, 80% of which resulted from non-compressible injuries (Behrens, Sikorski et al., 2014). In addition, the drug-induced effects, congenital or disease-related diseases, like clotting factor deficiency and platelet dysfunction, may also cause excessive bleeding (Demetri 2000; Blajchman 2003). There are two traditional methods concerning hemostatic treatment of external injury or lesion: compression hemostasis and drug pro-coagulation hemostasis. Both of them are effective in stopping bleeding, but they have some disadvantages, such as compression hemostasis being only suitable for vascular bleeding, and drug hemostasis can just be used as auxiliary means. In the case of non-compressible hemorrhage, either whole blood or blood components (red blood cells, plasma and platelets) are transfused (Kauvar, Holcomb et al., 2006; Spinella, Perkins et al., 2007; Chandler, Roberts et al., 2012; Kaufman, Djulbegovic et al., 2015; Spitalnik, Triulzi et al., 2015; Etchill, Myers et al., 2017), and fibrinogen concentrate or recombinant clotting factor (Berrettini, Mariani et al., 2001; Roberts 2001; Fries and Martini 2010; Rahe-Meyer and Sorensen 2011; Levy and Goodnough 2015) is used in some specific patients. However, sources for whole blood and its components are limited. Apart from this, the blood has a short shelf- life, a high risk of pathological contamination or immunogenicity, and limited portability. Therefore, developing new hemostatic biomaterials which can rapid hemostasis is a critical issue to improve hemodynamic stability and avoid the side effects of blood transfusion.

Understanding the mechanism of blood coagulation is the premise for hemostatic biomaterial. The process of blood coagulation contains two steps (Yang, Liu et al., 2017). Firstly, vasoconstriction, platelets adhere and aggregate to form a hemostatic plug, achieving preliminary hemostasis. And then, the blood coagulation cascade initiates plasma coagulation, and the fibrin network reinforces platelet thrombosis to achieve effective hemostasis in the second phase. Thus, the key to successful hemostasis is consisted by two parts: one is coagulation cascade, the other is platelet activation. In the coagulation cascade, activation of coagulation factor X affects thrombin generation, which directly determines the blood clotting. Platelet activation affects the formation of a hemostatic plug, it also activates prothrombin to achieve blood clotting.

Until now, a large number of hemostatic materials have emerged in the market. For example, α-cyanoacrylate and gelatin, which stop the bleeding by physically sealing the wound or compressing the blood vessels. One type of hemostatic biomaterial that creates a nanometer or micron-sized pore size, such as zeolites, mesoporous silica, etc. (Ellis-Behnke, Liang et al., 2006; Laurenti, Zazeri et al., 2017), and biopolymer materials such as gelatin and starch, which accelerates the initiation of physiological hemostasis by concentrating certain components of the blood under physical or chemical action and accelerating the activation of coagulation factors to stop bleeding. There are also hemostatic biomaterials that directly activates coagulation factors or platelets to activate the coagulation cascade (Gorbet and Sefton 2004), such as chitosan, alginic acid, oxidized cellulose and fibrin glue. The chitosan adheres to the wound to activate platelets and complement system in the blood. The alginic acid activates clotting factors after reacting with sodium ions. The oxidized cellulose can activate a variety of coagulation factors and aggregate platelets after rapidly dissolving in the blood, and the fibrin glue can rapidly initiate the endogenous coagulation system.

However, some hemostatic biomaterials have the following risks: 1) red blood cells may hemolyze and release hemoglobin, leading to anemia or renal failure, 2) plasma components such as platelets, white blood cells and complements in the blood may be activated to cause blood coagulation and inflammatory reactions at the same time (Motlagh, Yang et al., 2006). Therefore, a qualified hemostatic biomaterial should have the following characteristics (Kommu, McArthur et al., 2015): 1) Good biocompatibility, non-toxic, no antigenicity and inflammation; 2) suitable elasticity, good gas permeability and water permeability; 3) low infection probability, good tissue compatibility, and rapid hemostasis. In this review, various hemostatic biomaterials are summarized, containing the characteristics and clotting mechanism, development and application of the hemostatic biomaterials, strategies for optimizing the hemostatic biomaterials.

## The characteristic and application of the various hemostatic biomaterials

### Naturally derived biomaterials

In ancient times, people used herbs, greasy material and also sand mediated animal hides as hemostatic remedies. With the advancement in biotechnology, natural polymers have been introduced as hemostatic agents, which are naturally derived biomaterials that have good biocompatibility, mainly including protein-based hemostatic biomaterials, carbohydrate-based hemostatic biomaterials and inorganic hemostatic biomaterials. Protein-based hemostatic biomaterials include fibrin glue, collagen and gelatin, and carbohydrate-based hemostatic biomaterials include cellulose-based hemostatic biomaterials, chitosan, polysaccharides, and calcium alginate fibers; inorganic hemostatic biomaterials mainly include zeolite, kaolin, montmorillonite, and so on (Table 1).

**TABLE 1:** The different types of biomaterials for hemostasis.

Types	Materials	Brand names	Characteristics	Application
Protein-based	Collagen	Avitene^®^ Helitene^®^	Biodegradability, biocompatibility, cell adhesion, non-toxicity, and low antigenicity	It has a hemostasis in almost all operations, but cannot be applied to patients with thrombocytopenia
	Gelatin	Gelfoam^®^ Surgifoam^®^ Gelfilm^®^ Gelita-spon^®^	Has no toxicity or antigenicity, has soft fiber, strong adsorption, and can adhere to the wound surface	It could be used in the treatment of various wounds and traumas, in particular, transfixed wounds from solid organs. But it cannot be used in confined spaces or near nerve structures owing to easily causing complications of compressive origin
	Fibrin glue	Tisseel TachoSil^®^ Evicel^®^ Crosseal^®^	Good hemostatic properties, adhesive properties and histocompatibility	It can be applied to extensive bleeding after clinical tumor removal, bleeding in parenchymal organs, and patients with abnormal blood coagulation. But not allowed to be applied to blood vessels bleeding
Polysaccharide	Cellulose	Surgicel^®^ Original^®^ NuKnit Fibrillar SNoW^®^	Soft and thin, good histocompatibility, antibacterial effect	It is suitable for packaging, application, stuffing and other operations, hemostasis for capillary, arterioles and venous bleeding, bone bleeding. But it is not suitable for the treatment of peripheral nerve-rich wounds and irregular lacerations
	Chitosan	HemCon Celox Rapid gauze	Good affinity with human cells, no rejection, good biocompatibility, biodegradation, and hemostatic effect	It can be used in patients with coagulopathy. But hemostasis in the wound of extensive bleeding is not very satisfactory
	Starch	MPH, Per Clot	Reduce bleeding and transfusion, minimize blood infection, reduce seroma formation, has no immune, allergic reactions, and toxic side effects	It has effective hemostasis, but cannot enter into the blood vessel, lest form an embolism
	Alginate	Algosteril	Hygroscopicity, gelation, good biocompatibility, and the ability to ignite and shield electromagnetics	It is suitable for filling the wound, especially the deep and wide surgical cavity after endoscopic surgery
Inorganic	Zeolite, kaolin, montmorillonite	Quikclot, Woundstat Combat Gauze	Stability, ease of use, no biological toxicity, and no disease transmission	They cannot achieve rapid hemostasis, and are less effective in arterial bleeding and coagulopathic patients
Synthetic polymer-based	Poly(ethyelene oxide)	CoSeal^®^	Has good biocompatibility, histocompatibility, low cytotoxicity, and great hemostasis	It has been widely used in surgical bleeding
	Poly(cyanoacrylates)	Dermabond^®^ Omnex^®^ Glubran^®^and Glubran2^®^	Non-toxic, non-carcinogenic, and has good histocompatibility and significant hemostasis	It can be widely used in anastomosis, wound hemostasis, wound adhesion, and tendon. But may lead to vascular embolization and release toxic substances
Self-assembling peptide	RADA-16	SAPB-T45K, Pura Stat, Pura Matrix	Programmable, good viscoelasticity and biocompatibility, low antigenicity, and low cost	It can achieve complete and rapid hemostasis in the fields of the brain, spinal cord, liver, femoral artery and skin wounds
	SPG-178 PSFCFKFGP KOD			It overcame the disadvantage of RADA, whose low pH is easy to cause tissue damage. And it could coagulate whole blood, minimize bleeding

### Inorganic hemostatic biomaterials

**FIGURE 1:**
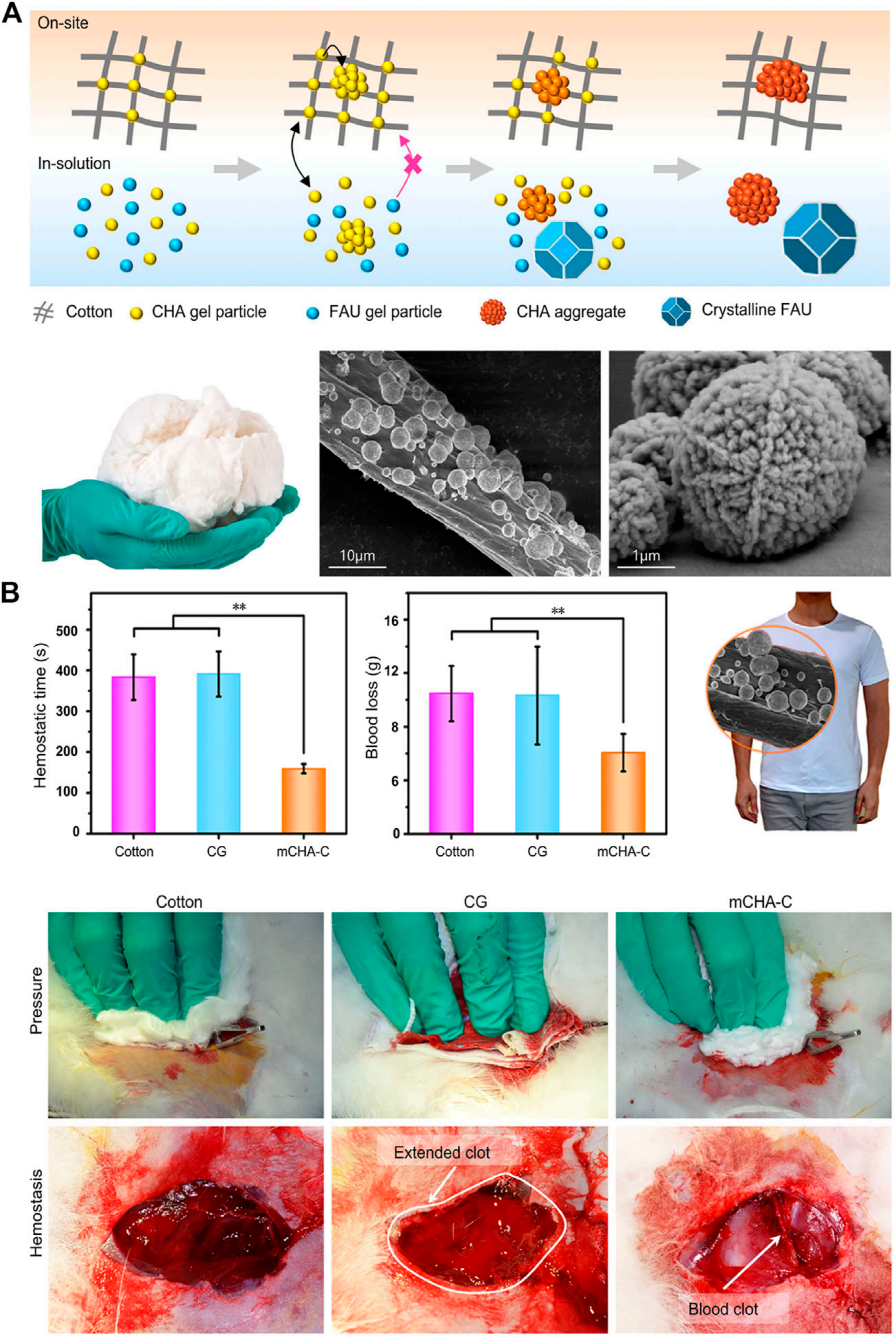
Inorganic biomaterials applied in hemostasis. **(A)** Upper:the schematic representation of formation process of on-site and in-solution products (Yu et al., 2019). Left: the photograph of mCHA-C. The SEM images of (Middle) mCHA-C, (Right) mCHA zeolite on cotton. **(B)** *In vivo* hemostatic capacity evaluation of hemostats. The quantitative analysis of hemostatic time (left) and blood loss (middle) in the rabbit femoral artery injury model. Right: Image of mCHA/T-shirt. The hemostasis was assessed upon manual pressure on the rabbit lethal femoral artery injury with cotton (left), CG (mid), or mCHA-C (right). Reproduced from (Yu et al., 2019) with permission from Copyright 2019 American Chemical Society.

**FIGURE 2:**
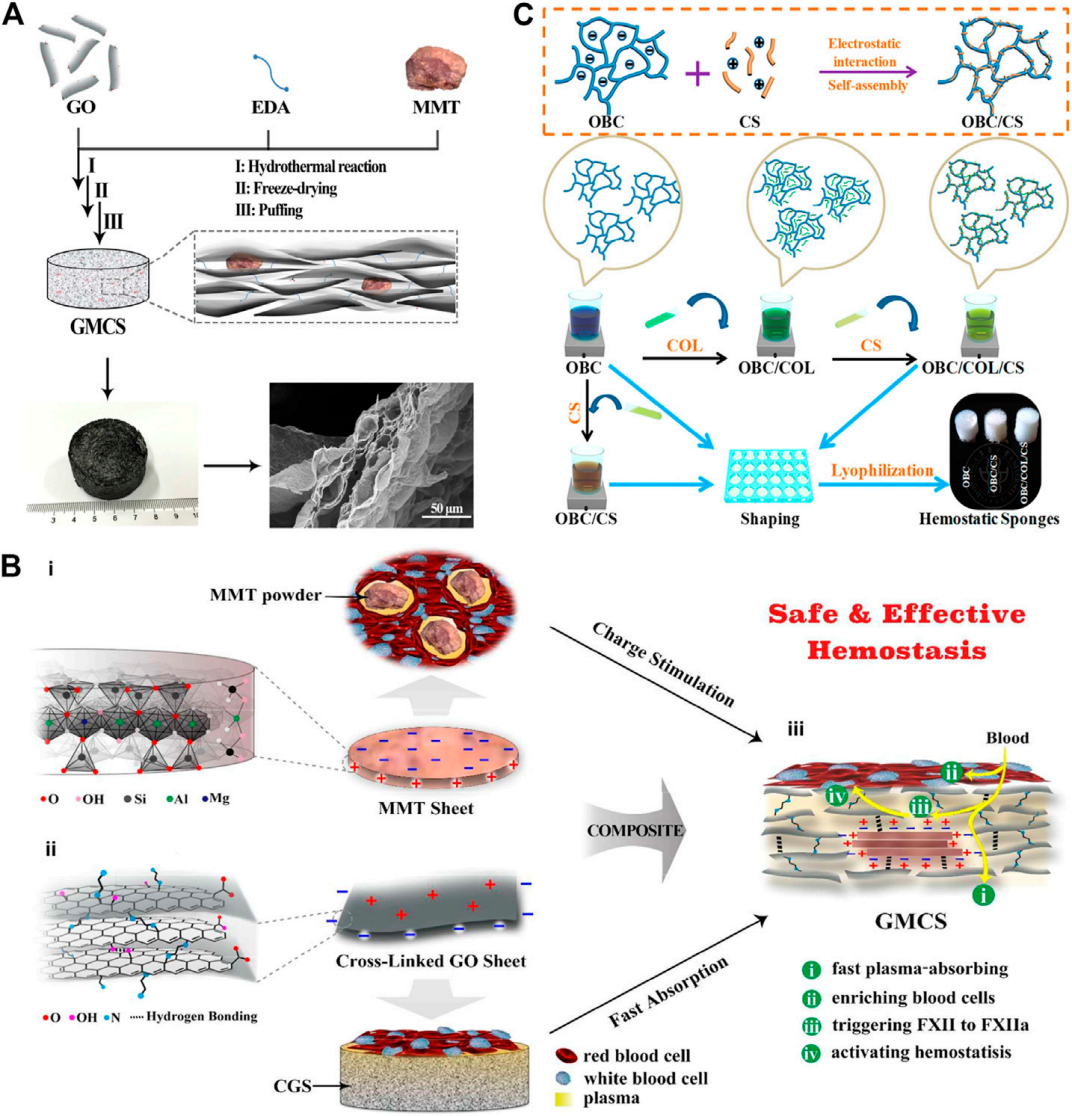
Naturally derived biomaterials are applied in hemostasis. **(A)** Schematic representation of the preparation route and the microstructure of the GMCS. The GMCS is firstly synthesized by hydrothermal reaction employing GO sheets, EDA linkers and MMT powders. Then, after freeze-drying and puffing treatments, the final product is obtained. The enlarged microstructure image shows MMT is fixed into the layered graphene. **(B)** Schematic representation of the GMCS construction and the potential synergy effect for hemostasis. (i) The MMT sheet possesses a permanent negative surface charge and a positive edge arising from the ordered and disordered crystal structure, respectively. Their powders can stimulate *in situ* clotting of blood with inward hydration and outward activating of blood coagulation. (ii) The cross-linked graphene sheet possesses a positive surface charge arising from EDA linkers and a permanent negative edge charge from inherent carboxyl groups. The resulted CGS can absorb plasma rapidly, increasing the concentration of hemocytes and platelets. (iii) MMT is fixed in the GMCS by the rich interactions, such as hydrogen bonding and electrostatic interactions. Reproduced from (Li et al., 2016) with permission from Copyright 2016 American Chemical Society. **(C)** Schematic diagram of the synthesis of OBC, OBC/CS, and OBC/COL/CS hemostatic sponges. Reproduced from (Yuan et al., 2020) with permission from Copyright 2019 American Chemical Society.

Zeolite (Yu, Shang et al., 2019), kaolin (Liang, Xu et al., 2018), montmorillonite (Li, Quan et al., 2016), etc. are all molecular sieve biomaterials, which have been applied to hemostasis. The zeolite has been approved by FDA as a topical hemostatic agent for clinical hemostasis. The main component of the first-generation Quikclot product is zeolite, which has an obvious exothermic effect of absorbing water when applied to the wound sites. Therefore, the research and development of early molecular sieve hemostatic materials are mainly focused on reducing its exothermic reaction, and developed the corresponding product, namely Quik Clot Sport Silver. Compared to other hemostats, they may cause more extensive blood loss due to failure to stop bleeding in a short time (Kheirabadi 2011; Pourshahrestani, Zeimaran et al., 2016). Moreover, long-term residual zeolite in the tissue may cause chronic inflammation. Hence, complete debridement should be conducted before wound closure. The researchers have been working on improving the hemostatic properties of zeolite by various methods and have found results. It was reported that mCHA-C (mesoporous zeolite CHA-cotton) has a better hemostatic effect by the mechanism that the gel particles induced on the cotton surface could be further developed to final fiber-bound CHA zeolite, or they self-assemble and crystalize into mesoporous CHA zeolitic crystals (Figure 1). Based on the biomaterials of montmorillonite and kaolin, the corresponding hemostatic products were developed, namely Wound stat and Combat Gauze. However, there are still many shortcomings, they cannot play a hemostatic role in arterial bleeding (Achneck, Sileshi et al., 2010; Granville-Chapman, Jacobs et al., 2011; Pourshahrestani, Zeimaran et al., 2016). Most importantly, all of these materials are less effective in coagulopathic patients, and can induce some problems, such as toxicity, tissue inflammation and embolization (Pourshahrestani, Zeimaran et al., 2016). Li, Quan et al. (2016) have developed a graphene-MMT composite sponge (GMCS) to solve these problems (Figure 2A). It was confirmed that it has good hemostasis through these aspects (Figure 2B): 1) fast-absorbing of plasma within the sponge; 2) concentrating blood on the sponge surface; 3) activating clotting factors by MMT; 4) accelerating the speed of blood clotting, totally resulting in an ultrafast hemostasis.

In addition, both of protein-based and polysaccharide biomaterials have their own advantages on hemostasis. The researchers have developed a OBC/CS (oxidized bacterial nanocellulose/Chitosan) and OBC/COL/CS (oxidized bacterial nanocellulose/Collagen/Chitosan) sponge and confirmed the role of hemostasis in a rat liver trauma model (Yuan, Chen et al., 2020) (Figure 2C).

### Protein-based hemostatic biomaterials

#### Fibrin glue

Currently, there is a wide variety of fibrin sealants on the market, most of which are composed of thrombin and purified human or bovine fibrinogen, factor XIII, to mimic the physiological coagulation process (Watrowski, et al., 2017). Absorbable fibrin glue consists of fibrinogen, thrombin, aprotinin and calcium chloride, which is the most effective and widely used fibrin sealant in hemostasis. Fibrin glue has some excellent properties, such as good hemostatic properties, adhesive properties and histocompatibility, which effectively reduces the amount of blood loss and blood transfusion during the operations. At present, fibrin glue has been applied to extensive bleeding of wounds after clinical tumor removal and bleeding in parenchymal organs such as liver and kidney. In addition, since it contains thrombin, fibrin glue can be applied to patients with abnormal blood coagulation. However, due to its adhesion and procoagulant effect, fibrin glue is not allowed to be applied to blood vessels bleeding in case the formation of blood clots blocks blood vessels. In addition, due to its complicated procedure and inconvenient storage and transportation, fibrin glue is also not suitable for emergencies. Moreover, fibrin glue also has some disadvantages, such as the high cost, the potential for infecting human or animal hematogenous diseases (Valeri 2006), and the low efficiency of hemostasis for great vessels, limiting its application range.

#### Collagen

Collagen has some excellent performances, such as good biodegradability, biocompatibility, cell adhesion, non-toxicity, and low antigenicity (Hu, Yu et al., 2017). As a hemostatic biomaterial, collagen is always made into a porous or fibrous sponge which is beneficial for hemostasis of various wounds and the occurrence of hemostasis in almost all operations. A collagen sponge is especially applied to local bleeding where it is difficult to perform ligation during surgery, bleeding in fragile tissue or blood vessel-rich parts, and oozing of large areas in soft tissue. However, because the hemostasis of collagen requires the activation of platelets (Lewis, Kuntze et al., 2015), it cannot be applied to patients with thrombocytopenia, but it still has a good hemostasis effect on hemorrhage caused by heparinization. Overall, collagen not only has the disadvantages of low mechanical strength, uncontrollable biodegradation rate, variability, potential pathogenic risk, etc. it is also a foreign protein, which increases the chance of infection and allergic reactions (Seyednejad, Imani et al., 2008). Excessive swelling can result in nerve compression and damage (Achneck, Sileshi et al., 2010). These shortcomings limit the use of collagen as a hemostatic biomaterial.

#### Gelatin

Gelatin-based biomaterial is not only absorbant by tissue cells, but also has no toxicity and antigenicity, and does not cause excessive scar tissue or cellular reactions. Hence, it could be used in the treatment of various wounds and traumas, in particular, transfixed wounds from solid organs. But it cannot be used in confined spaces or near nerve structures owing to the fact that it could easily cause complications of compressive origin (Pereira, Bortoto et al., 2018). The gelatin sponges and gelatin fibers are most common in hemostasis. A gelatin sponge (GS) is a porous sponge that could absorb blood about 45 times heavier than itself (Piozzi, Reitano et al., 2018). Moreover, the porous structure of the gelatin foam is conducive to the aggregation and proliferation of fibroblasts, which then form new granulation tissue along the gelatin foam and rapidly fill the cavity and sinus tract. However, the gel formed by the gelatin sponge and the blood is soluble, so the above-mentioned hemostatic state and structure are easily broken. Moreover, it also has a poor traction ability on hemostatic components such as platelets and adhesion ability on the wound surface. Therefore, gelatin foam has a poor effect on hemostasis.

### Polysaccharide hemostatic biomaterials

#### Cellulose

Oxidized cellulose (OC) and oxidized regenerated cellulose (ORC) are topical hemostatic biomaterials that have the appearance and texture of cotton yarns and are bioabsorbable. Both of them are soft and thin and have good histocompatibility, suitable for packaging, application, stuffing and other operations. When in close contact with the wound, the clotting components of the blood can be gathered around it, and hemostasis can be completed within 2–8 min. The soluble hemostatic gauze (Surgice1), which is often used in the clinic, is a regenerated oxidized fiber woven yarn, and belongs to the carboxymethylcellulose hemostatic material. Due to its slow dissolution, it is generally absorbed within 3–6 weeks, and is often used for bleeding on surgica1 wounds and sites where bleeding cannot be stopped, such as bone bleeding, etc. Moreover, it can play an effective role in hemostasis for capillary, arterioles and venous bleeding (Tam, Harkins et al., 2014). In addition, the Surgicel can lower the local pH of the wound to acidic, which has a certain antibacterial effect (Masci, Faillace et al., 2018).

However, the premise of using such hemostatic biomaterials is that the patient must have a complete coagulation function. In the absence of coagulation factors, the role of such hemostatic biomaterials in activating platelets is significantly weakened. Apart from this, Nagamatsu et al. (1997) found in the study of the formation of neuropathy that the highly acidic environment generated by oxidative cellulose can cause nerve injury through a diffuse chemical mechanism. The improved oxidized cellulose has a better hemostatic effect and fewer adverse reactions, but it is still not suitable for the treatment of peripheral nerve-rich wounds.

#### Chitosan

Chitosan is a primary derivative of chitin deacetylation and is a rare alkaline polysaccharide in nature. Chitosan has excellent properties, such as good affinity with human cells, no rejection, good biocompatibility, biodegradation, and has a hemostatic effect (Chan, Kim et al., 2016). It also has the following characteristics: 1) promote the secretion of hyaluronic acid and other glycosamines, 2) accelerate wound healing, 3) inhibit the growth of a variety of bacteria and fungi, 4) increase the mechanical properties of biomaterials, etc. (Pusateri, McCarthy et al., 2003). Therefore, a variety of medical materials such as chitosan-free gauze, chitosan-coated gauze and chitosan hemostatic sponges are used in clinical practice. The coagulation mechanism of chitosan is independent of blood clotting factors and platelets, so it can be used in patients with coagulopathy. However, due to the limited effect of hemostasis of chitosan, it is not very satisfactory in the wound with extensive bleeding. Therefore, the method of compounding other hemostatic agents, such as clotting factors and calcium chloride, are often used.

#### Starch

MPH (Microporous polysaccharide hemispheres) and Per Clot are hemostatic products of microporous polysaccharides prepared by extracting from plant starch and further processing. MPH containing no polypeptide or protein can not only rapidly dehydrate blood to form blood clots to prevent blood from oozing out, but also can be rapidly degraded and absorbed *in vivo*. At the same time, due to the low protein property of MPH, the local tissue response is weak, rarely causing foreign body reactions and reducing the risk of infection during use (Humphreys, Castle et al., 2008). As a hemostatic agent, the microporous polysaccharide has its own advantages: 1) effectively reduce the amount of bleeding and transfusion; 2) minimize the rate of blood-derived infection; 3) reduce seroma formation; 4) it has no immune, allergic reactions, and toxic side effects on wound healing (Egeli, Sevinç et al., 2012). Morover, these biomaterials cannot enter the blood vessel, lest form embolism.

#### Calcium alginate fiber

Calcium alginate fiber is a fibrillar-like polysaccharide, which is extracted from seaweed. It has the properties of hygroscopicity, gelation, good biocompatibility, and the ability to ignite and shield electromagnetics. Due to its excellent adhesion, it is suitable for filling the wound, especially the deep and wide surgical cavity after endoscopic surgery. Importantly, the formed gel material will not adhere to the operative cavity and is conducive to repairing nasal mucosa epithelium.

### Synthetically derived hemostatic biomaterials

Synthetic hemostatic agents are typically made from cyanoacrylate, polyurethane, and polyethylene glycol. Because they have low immunogenicity and can customize their chemical properties to stimulate procoagulant mechanisms, they are widely used in various hemostasis operations. However, the toxic by-products from these hemostatic agents aggregate and degrade, causing local irritation and inflammation (Table 1).

### Polymers-based hemostatic biomaterials

#### Poly(ethyelene glycol)

The polyethylene glycol (PEG), a synthetic polymer, is soluble in both aqueous and organic solvents, therefore it can interact with both the intra- and extra-cellular spaces, which provides an environment beneficial to cellular infiltration and growth (Harris 1992). It has been widely used in biomedical engineering, such as regeneration of nerves, articular cartilage and bladder tissue (Yamawaki, Taguchi et al., 2017).

There are some advantages, for example, completely no risk of infectious diseases, batch-batch stability, low price, and can be prepared in batches. In addition, a hemostatic liquid sealant made of the tetra-succinimidyl -derivatized PEG and tetra-thiol-derivatized PEG could stop bleeding by crosslinking with tissue and sealing the hemorrhagic spot (Overby and Feldman 2018). The commercial product, namely AdvaSeal^®^, was also developed by co-polymerizing poly(ethylene glycol) with poly(α-hydroxy acid) diacrylate. Moreover, the poly(ethyelene oxide) based hemostatic biomaterials have been widely used in surgical bleeding, such as Poly(alkylene oxides), e.g., poly(ethylene oxide) (PEO) and poly(propylene oxide) (PPO). For instance, Wang et al. and Wellisz et al. have reported that a PEO-PPO-PEO block copolymer-based waxy material, namely Ostene^®^, has an excellent effect on hemostasis in orthopedic surgeries (Hickman, Pawlowski et al., 2018).

#### Poly(cyanoacrylates)

Although α-cyanoacrylates have tissue toxicity at a low level, some of them have been widely used in the clinic, such as n-cyanoacrylate, n-butyl α-cyanoacrylate, n-octyl α-cyanoacrylate. And it is proven that they are non-toxic, non-carcinogenic, and have good histocompatibility and significant hemostasis. It was reported that the Omnex^®^ consisting of n-octyl α-cyanoacrylate and butyl lactoyl-2-cyanoacrylate could stop bleeding (Bhatia 2010). In addition, the Glubran^®^ and Glubran2^®^, which are made of n-Butyl-2-cyanoacrylate and methacryloxysulpholane, have bacteriostatic and hemostatic properties (Montanaro, Arciola et al., 2000). For wounds with major artery bleeding, a tourniquet is first placed on proximal vein to temporarily stop bleeding, then the α-cyanoacrylate tissue glue is sprayed on the wound after wiping the blood. It is an ideal hemostatic method that has a high success rate of hemostasis and the side effects are minor (Fontenot, Rasmussen et al., 2005). At present, α-cyanoacrylate can be widely used in the anastomosis, wound hemostasis, wound adhesion, and tendon (de Azevedo, Marques et al., 2003; Lumsden and Heyman 2006). However, such hemostatic materials can lead to vascular embolization and release toxic substances such as cyanide and formaldehyde in the degradation process, which may induce inflammation and tissue necrosis around the injection site (Farooq and Wong 2005).

### Self-assembling peptide as hemostatic biomaterials

Under physiological conditions, Self-assembled peptides can spontaneously and regularly form stable secondary structures through non-covalent bonds, then further stacked into nano-fiber to form a hydrogel scaffold structure with a water content of more than 99%. The self-assembled peptides have excellent performances, such as programmable, good viscoelasticity, good biocompatibility, low immunogenicity and low cost (Kondo, Nagasaka et al., 2014; Rad-Malekshahi, Lempsink et al., 2016; Gao, Xu et al., 2017). Moreover, without the help of traditional hemostatic methods, such as pressure, cautery, vasoconstriction, coagulation and cross-linking agent, it can achieve complete and rapid hemostasis in the fields of the brain, spinal cord, liver, femoral artery and skin wounds (Fouani, Nikkhah et al., 2019).

**FIGURE 3:**
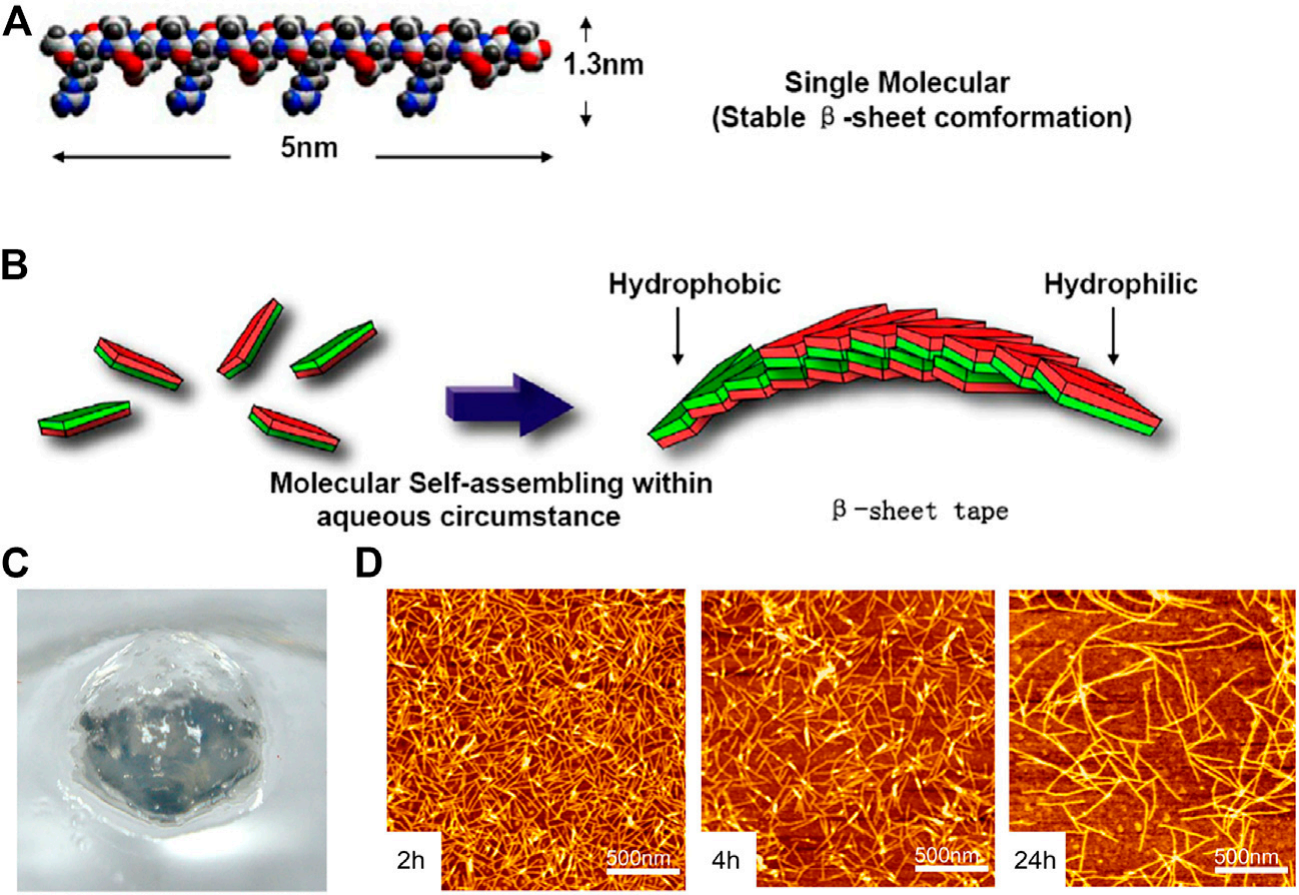
The RADA16 is applied in hemostasis. **(A)** Molecular model of the RADA16-1; **(B)** Molecular model of numerous RADA16-1 as they undergo self-assembly to form nanofibers with the hydrophobic alanine sandwiched inside and hydrophilic residues on the outside; **(C)** RADA16-1 gelatinized into hydrogel. Reproduced from (Wang et al., 2012) with permission from Copyright 2012. **(D)** AFM images of RADA16-I nanofibers at various points in time after sonication. Note the elongation and reassembly of the peptide nanofibers over time.

RADA16-I (Ac-RADARADARADARAD-NH_2_) is thoroughly studied and widely used as a self-assembling hemostatic biomaterial. The monomer of RADA16-1was ≈5 nm long, ≈1.3 nm wide, and ≈.8 nm thick in dimensions (Figure 3A). RADA16-1 samples can spontaneously assemble nanofibers ranging from a few hundred nanometers to a few microns in length (Figure 3B) and formed hydrogels that were achromatic color, hyaloid and can be fabricated into various geometric shapes (Figure 3C). The results of AFM (Wang et al., 2012) showed that RADA16-1 could form long self-assembled nanofibers ranging with hundred nanometers in a stable and repeatable manner (Figure 3D). Masuhara, Fujii et al. (2012) confirmed the hemostatic effect of RADA16-I by rabbit abdominal aortic puncture bleeding model, and also tested the safety of intravenous injection of lower concentration hydrogel. It has a 3D structure similar to ECM (extracellular matrix), which facilitates cell adhesion, proliferation and differentiation (Guo, Wang et al., 2011; Mie, Oomuro et al., 2013; Gao, Xu et al., 2017), and the European-certified commercial product which contains this biomaterial named Pura Stat is applied in clinical hemostasis. It was reported that RADA not only prevented traumatic bleeding in the brain, spinal cord, femoral artery and liver (Ellis-Behnke and Schneider 2011), but could also rapidly achieve hemostasis in both heparinized and non-heparinized animals, solving a series of problems caused by the use of anticoagulant drugs in surgical intraoperative and postoperative bleeding (Csukas, Urbanics et al., 2015).

Komatsu, Nagai et al. (2014) designed a neutral (pH = 6.5–7.5) self-assembled peptide hydrogel namely SPG-178 (RLDLRLALRLDLR), which could achieve rapid hemostasis in the left hepatic lobe. Ruan, Zhang et al. (2009) designed an amphiphilic self-assembled short peptide (PSFCFKFGP), which was mainly self-assembled as a spherical polymer at a high concentration and then passed through the nanofiber body to form a hydrogel with a water content of more than 99%. And the results of the comparative experiment on hemostasis showed that the hemostatic time of the 1% short peptide hydrogel group was shorter than the gauze group and chitosan group. Kumar, Taylor et al. (2014) designed a mimetic peptide of collagen named KOD ((PKG)4(P-Hyp-G)4(D-Hyp-G)4), consisting of 36 amino acids. The KOD can self-assemble into three helical nanofibers to further form nanofiber hydrogels, which have been shown to coagulate whole blood, minimize bleeding, and significantly activate platelets.

The self-assembled peptides have some hemostatic properties: 1) when contacting with electrolytes in body fluids, self-assembled peptides begin to self-assemble and form a reticular hydrogel structure with a pore size of 50–200 nm, which can quickly fill and match irregular wounds, then form a nanofiber barrier to prevent the exudation of cells and body fluids. 2) High-concentration short peptides can self-assemble more nanofibers, better facilitating blood coagulation. Moreover, ions and charges can accelerate the self-assembly of short peptides into nanofibers in the blood containing red blood cells (Luo, Wang et al., 2011). 3) The self-assembled peptide hydrogel is a colorless and transparent biological scaffold, which could clearly observe the hemostasis inside the wound. 4) The elastic modulus of self-assembled peptide hydrogel is either too high or too low to achieve the best hemostatic effect. Only when the short peptide hydrogel closely fits the wound can it better withstand the vascular pulse and exert the maximum hemostatic effect. 5) The degradation products of self-assembling peptide hydrogels are natural L amino acids that can be absorbed by surrounding tissues for repair. Therefore, one of the hot topics is to develop a variety of self-assembling peptides with antimicrobial properties and hemostatic effects.

## The mechanism of the hemostatic biomaterials

**FIGURE 4:**
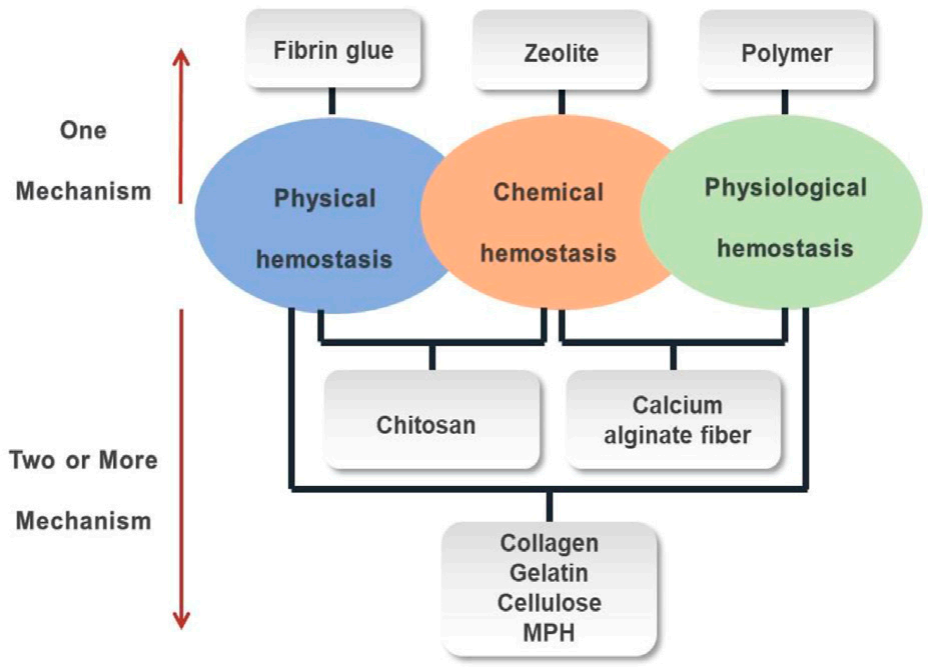
The hemostatic biomaterials with different mechanisms.

There are three hemostatic mechanisms when the most functional hemostatic biomaterials are exposed to blood (Figure 4). 1) Physical hemostasis: the biomaterial absorbs water in the blood, causing the viscosity and concentration of the blood to increase, thereby slowing down blood flow; or the biomaterial swells after absorbing water to cover the wound surface and stopping the bleeding. 2) Chemical hemostasis: the presence of negative ions can quickly bind or aggregate red blood cells and platelets, thereby releasing blood coagulation-related factors to accelerate blood coagulation. 3) physiological hemostasis: the biomaterial can rapidly activate coagulation delivering factors II, V, VII, X, and XII, which activates the endogenous coagulation system and forms insoluble fibrin polymer with thrombin to achieve hemostasis.

Some hemostatic biomaterials achieved the purpose of hemostasis through a mechanism, such as zeolite, fibrin glue and polymer. The hemostatic mechanism of zeolite is to concentrate platelets and clotting factors through the rapid absorption of water at the bleeding site, while the heat generated by water absorption enhances the rate and ability of aggregation of platelets, significantly improving the survival rate of patients with large areas and severe trauma. The fact that zeolite does not contain any biological components has some advantages, such as stability, ease of use, no biological toxicity and no disease transmission (Li, Cao et al., 2013). The hemostatic mechanism of fibrin glue is that thrombin cleaves the fibrinogen into fibrin, which causes blood coagulation, while aprotinin inhibits the dissolution of blood clots, making blood clots more stable on the wound, thereby achieving hemostasis. This polymer hemostat achieved the purpose of hemostasis through a tamponade mechanical effect, not a biochemical augmentation of coagulation mechanisms. They do not have a hemostatic effect by themselves, but could achieve hemostasis through physical closure, mechanical barriers, and wound closure.

In addition, the hemostatic biomaterials achieved the purpose of hemostasis by more than one mechanism, such as collagen, gelatin. Collagen mainly has a hemostatic function through the following three aspects: 1) activating partial coagulation factors to produce thrombin which cleaves fibrinogen into fibrin (Skopinska-Wisniewska, Sionkowska et al., 2009), 2) aggregating platelets to form a thrombus, preventing bleeding, 3) adhering to the wound and mechanically compressing the damaged blood vessel to serve as a packing effect. The gelatin, as a physical matrix, affects clot initiation. When it is in contact with blood, the gelatin would swell and induce a buffering effect. The hemostatic mechanism of a gelatin sponge is relatively simple, that is, it provides an attachment surface for platelets and stimulates the release of platelet factors. Gelatin fiber (GF) is a non-woven hemostatic biomaterial made from a gelatin sponge and extracts of a maple leaf, which can promote platelet adhesion and aggregation, and then accelerate hemostasis. Furthermore, the gelatin fiber has a dense network structure with uneven loose space inside, which enlarges the contact area with blood, so that the platelets are more likely to adhere and accumulate on the fibers, which is conducive to the formation of white thrombus and then achieve rapid hemostasis. Because gelatin fiber has strong adsorption, and can adhere to the wound surface, it has a more obvious hemostatic effect than a gelatin sponge in the deep part of the body.

The Polysaccharide hemostatic biomaterials achieved the purpose of hemostasis by different mechanismsm, such as cellulose, chitosan, MPH and calcium alginate fiber. Both oxidized cellulose and oxidized regenerated cellulose achieve the purpose of hemostasis in two aspects: on the one hand, a viscous gel block is formed by combining an acidic carboxyl group with Fe^3+^ in hemoglobin, to seal the damaged capillaries and stop bleeding; on the other hand, it activates the body’s coagulation mechanism by adhering and accumulating platelets, to accelerate blood clotting. It was (Bano, Arshad et al., 2017) reported that positively charged molecules in chitosan could combine with negatively charged tangible components in blood, such as red blood cells, white blood cells and platelets, to form cell emboli or thrombus to play a role in blood coagulation. Particularly, due to the hydrophilicity of the amino group in the chitosan, more fibrinogen is adsorbed, thereby promoting the formation of a thrombus. The mechanism of MPH includes two aspects. One is that when the biomaterial is placed on the bleeding wound, the particles quickly absorb the water in the blood to concentrate the components in the blood such as platelets, red blood cells, and clotting factors (Thongrong, Kasemsiri et al., 2013), to form a gelatinous mixture, which plays a role on the instant hemostasis. Another is that the biomaterial accelerates the activation of endogenous clotting factors and forms a strong topical clot, shortening the clotting time (Ersoy, Kaynak et al., 2007). The calcium alginate fiber has been reported to have great hemostasis (Aydin, Tuncal et al., 2015). When the calcium alginate fiber is in contact with the sodium salt in the blood and wound secretion, it can absorb 15–20 times of blood than its weight and is converted into a gel substance, and simultaneously releases calcium ions to stop bleeding.

## Hemostatic strategies in biomaterials

### Optimization of the single hemostatic biomaterial

Various hemostatic materials have been emerging in recent years, but it is still a great challenge to achieve hemostasis in surgery. Different types of hemostatic biomaterials has their own shortcomings, which affects the efficacy of hemostasis. For instance, although the fibrin sealants could effectively enhance blood coagulation, they still have a potential risk of spreading viral infections. The acidic nature of cellulose products may have a side effect on the surrounding tissue and wound healing. The collagen-based hemostatic agents are easy to cause allergic reactions, as well as nerve compression and damage, resulting from excessive swelling. Inorganic hemostats may elicit a foreign-body response due to their poor biodegradability. Thus, many researchers are now committed to the optimization of biomaterial properties. The optimum methods for a hemostatic biomaterial are that 1) could change the mechanical properties by cross-linking technology, etc., to make the biomaterial more suitable for hemostatic application; 2) could optimize the nucleic sequence of certain protein by genetic engineering technology and recombinantly expressed to obtain a novel protein, which possesses new excellent properties and could overcome the inherent shortcomings of the biomaterial.

### Modification of the biomaterial by crosslinking

It is necessary to modify the material intrinsically to overcome some shortcomings of the material. Taking collagen as an example, although collagen-based hemostatic biomaterials have great advantages. The disadvantage of the simple collagen limits its hemostatic application, such as poor mechanical properties, uncontrollability of biodegradation rate, variability, etc. Nowadays, cross-linking techniques are commonly used to change the physical properties of collagen. The method of physical cross-linking is simple, and has low cytotoxicity, mainly including hot-dry cross-linking (DHT) and ultraviolet cross-linking.

However, compared with physical cross-linking, chemical cross-linking has a better control of structure and could improve some properties of collagen, such as tensile strength, flexibility, mechanical properties, biodegradation rate and thermal stability. The main chemical cross-linking agents include aldehydes, dicarboxylic acids, genipin, carbodiimide, citric acid derivatives, chitosan and polyvinyl alcohol. After modifying the tilapia skin collagen sponge through chemical cross-linking methods (Verissimo, et al., 2010; Xie, Yi et al., 2018), the sponge has shown more excellent performances, such as mechanical properties, the elongation at break, and the collagenase degradation. Thus, it was an ideal medical hemostatic material with biocompatibility. It was reported that the collagen-cotton meshed by chemically modifying soluble collagen with aldehydes improved the hydrophilicity of collagen and its hemostatic performance. Chen, Zhang et al. (2016) designed the amphiphilic short peptide called I3QGK, which combines the self-assembly process of short peptides with the catalysis of transglutaminase (TGase), and was applied to hemostasis. It could form the hydrogel with good mechanical properties, and can effectively stop bleeding by gelatinizing blood and promoting platelet adhesion. The development of cross-linking technology is also very important. Although chemical cross-linkers can form strong bonds, most of them are cytotoxic. Therefore, the quality and biocompatibility of collagen hemostatic sponge prepared with chemical cross-linkers are often unsatisfactory. At present, the method of enzyme cross-linking, which has a milder condition and good biocompatibility, is commonly used in the fabrication of hemostatic biomaterials.

### Genetic engineering for optimizing biomaterials

The cross-linking techniques can change certain properties of biomaterials to make them more suitable for hemostasis, but they cannot eliminate all the disadvantages of biomaterials. For example, since natural collagen is mostly extracted from animals, it has the potential of pathogenic transmission and the disadvantage of batch-batch instability. However, the proteins expressed by genetic recombination can overcome the above shortcomings, and can also realize large-scale production of proteins at a low price. Therefore, based on optimizing the nucleic acid sequence of natural collagen, human-like collagen (Fan, Luo et al., 2005) was obtained through genetic recombination technology, which has good biocompatibility and biodegradability. Based on the above, a new styptic sponge was prepared by cross-linking human-like collagen with glutamine transaminase and a two-step freezing method (Jiang, Wang et al., 2017), which has good biocompatibility and a significant hemostatic effect in the ear artery model and liver injury model.

### Creation of the composite hemostatic biomaterials

For certain biomaterials, it is possible to strengthen the properties of biomaterial through cross-linking technology or genetic recombination technology in order to stop the bleeding more effectively. Based on this assumption, it may be better to combine two or more kinds of biomaterials to strengthen advantages and avoid disadvantages, and thus achieve the purpose of more rapid and effective hemostasis (Table 2).

**TABLE 2:** The summary of composite hemostatic biomaterials.

Types	Major components	Advantages	References
Sponges	Collagen/chitosan/calcium pyrophosphate	It not only enhances the hemostatic effect, but also solves the problem of cavity filling in surgery	Yan, Cheng et al. (2017)
	Chitosan/gelatin	It has good biodegradability, no cytotoxicity, effective bacteriostasis, and could promote cell proliferation	Lan, Lu et al. (2015)
	Silk fibroin/gelatin	It significantly reduces the wound area and promotes wound healing, the formation of skin and collagen	Harrison and Spada (2018)
Films	Silk fibroin/polylactide (1:2)	As a hemostatic material for chronic wound	Shahverdi, Hajimiri et al. (2014)
	Chitosan/Ga_2_O_3_-containing mesoporous bioactive glass	It could promote cell proliferation, and has excellent antibacterial activity, high hemostatic efficacy	Ostomel, Shi et al. (2006), Dai, Yuan et al. (2009), Salinas, Shruti et al. (2011), Shruti, Salinas et al. (2013), Shruti, Salinas et al. (2013)
Hydrogels	Poly(vinyl alcohol)/human-like collagen/carboxymethyl chitosan	It has excellent swelling ratios, bacterial barrier activity, moisture vapor permeability, hemostasis activity and biocompatibility	Pan, Fan et al. (2017)
	Dihydroxyacetone (pDHA)/methoxypoly(ethylene glycol) (MPEG)	It eliminates the major problem of the insolubility of pDHA, and has some advantages, such as short residence time *in vivo*, low inflammation, and the risk of infection	Henderson, Kadouch et al. (2010)
	GelMA/HA-NB	The GelMA/HA-NB matrix hydrogel could adhere to the wet wound tissue and seal the hemostatic site through UV light irradiation	Yang, Zhang et al. (2016) and Hong, Zhou et al. (2019)
	SLac/bactretase	The SLac in itself provides a physical barrier to bleeding wounds. And SLac loaded with bactretase produced a kind of drug-loaded hydrogel (SB50), which enhanced the role of coagulation	Kumar, Wickremasinghe et al. (2015)
Fibers	Chitosan/recombinant batroxobin	It has a synergetic effect on blood coagulation	Seon, Lee et al. (2018)
	Chitosan/geltain	It has high porosity, rapid blood absorption, and yields synergistic effects in hemostatic function and wound repair	Gu, Park et al. (2016)
Particles	Chitosan/poly(vinyl alcohol)	It effectively reduces thromboembolic formation in stopping bleeding from femoral artery	Chen, Liu et al. (2017)
	Chitosan/Kaolin clay	It has high amount of pores, no adverse effects, and the synergetic combination mechanisms	Sun, Tang et al. (2017)
	A mesoporous silica sphere doped with calcium-silver	It has good degradability and antibacterial properties	Dai, Yuan et al. (2009)
liquid	tetra-succinimidyl-derivatized poly(ethylene glycol)/tetra-thiol-derivatized poly(ethylene glycol)	stop bleeding by cross-linking with tissue and sealing the hemorrhagic spot	Overby and Feldman (2018)
	Gelatin based bovine collagen/microgranules/glutaraldehyde/human thrombin	It is superior to Gelfoam-thrombin in cardiac surgeries, and has reduced bleeding when used in open nephrectomies and laparoscopy	Reuthebuch, Lachat et al. (2000) and Oz, Rondinone et al. (2003)
	ABS/(Lauryl -VVAGK-Am)	It has an excellent hemostatic effect in partial nephrectomy	Huri, Dogantekin et al. (2016)
Powder	Hydroxyethylcellulose/EGF (CEGP-003)	It appears to have effective treatment in peptic ulcers and ESD- or EMR-induced bleeding	Bang, Lee et al. (2018)

The Yan, Cheng et al. (2017) designed a novel collagen sponge combined with chitosan/calcium pyrophosphate for hemostasis. It has dual hemostatic effects: on the one hand, collagen adsorbs platelets and activates blood coagulation factors, and compresses small blood vessels to stop bleeding; on the other hand, the hemostatic mechanism of chitosan is that it promotes the aggregation of red blood cells to form a red thrombus. Furthermore, it not only enhances the hemostatic effect, but also solves the problem of cavity filling during in surgery. The pH of the gelatin matrix is neutral, so it is used in conjunction with other hemostatic agents, such as biomaterials that blend chitosan with gelatin. These biomaterials have the advantages of the chitosan, such as good biodegradability, biocompatibility, antibacterial and film-forming properties, as well as the advantages of gelatin, such as low antigenicity, etc. The Guangqian Lan, Lu et al. (2015) prepared a kind of composite containing chitosan and gelatin for hemostasis by cross-linked with tannins and then freeze-dried under a vacuum. A double-layer hemostatic biomaterial (Harrison and Spada 2018), which was fabricated by combining silk fibroin and gelatin, has been reported. The silk fibroin fabric was used as a non-adhesive layer to contact the wound, while the bioactive layer of the silk fibroin/gelatin sponge could significantly reduce the wound area, promote wound healing and the formation of skin and collagen. Shahverdi, Hajimiri et al. (2014) optimized the electrostatic spinning process for a mixture of silk fibroin/polylactide (1:2) to make the mixed membrane as a hemostatic biomaterial for a chronic wound. Due to the unique features of mesoporous bioactive glasses (MBGs), such as high porosity, highly ordered mesoporous channel structure, huge surface area and pore volume (Ostomel, Shi et al., 2006; Dai, Yuan et al., 2009; Salinas, Shruti et al., 2011; Shruti, Salinas et al., 2013; Shruti, Salinas et al., 2013), it has excellent hemostatic property, as shown by enhancing capability of platelet aggregation, thrombus formation, and blood coagulation activation. Based on the above, some researchers also reported (Pourshahrestani, Zeimaran et al., 2017) that a 1% Ga-MBG/chitosan (Ga-MBG/CHT) composite scaffold has a rapid hemostatic effect. The researchers (Pan, Fan et al., 2017) have prepared a novel hemostatic biomaterial through simply repeated freeze-thawing of the mixed solution including poly(vinyl alcohol), human-like collagen and carboxymethyl chitosan, which showed a great hemostasis effect. The potential of poly(dihydroxyacetone) (pDHA) in hemostatic application is limited owing to its insolubility in all aqueous and almost all organic solvents. It was reported (Henderson, Kadouch et al., 2010) recently that a kind of block copolymer made of PEG and pDHA could be an effective and rapidly resorbable hemostatic agent, which has some advantages over other hemostats, such as short residence time *in vivo*, low inflammation and the risk of infection. It was reported (Seon, Lee et al., 2018) that an efficacious hemostatic agent made of chitosan-based non-woven dressing with recombinant batroxobin (rBat), which has a synergetic effect on blood coagulation. In addition, some researchers found (Gu, Park et al., 2016) that sonicated chitosan-geltain nanofiber mats with high porosity yield a synergistic effect in some ways, including hemostatic function and wound repair. Dai, Yuan et al. (2009) prepared hemostatic biomaterials doped with calcium-silver mesoporous silica spheres, and the results showed that the calcium ions in the biomaterials improved hemostatic properties. Poly(vinyl alcohol) (PVA) was conducive to increasing the swelling degree of the spheres and the enhanced hemostatic effect. To take full advantage of the PVA and chitosan, the Chitosan-PVA spheres (Chen, Liu et al., 2017) were prepared through electrospraying and ionotropic gelation, which effectively reduced thromboembolic formation in stopping the bleeding from the femoral artery. Sun, Tang et al. (2017) fabricated porous chitosan/kaolin composite microspheres (CSMS-K), whose hemostatic performance was superior to chitosan porous microspheres (CSMS) that it formed larger blood clots than CSMS and Celox within the same time period. The hemostat called FloSeal^®^, which is a gelatin matrix based on bovine collagen containing micro granules, crosslinked with glutaraldehyde and human thrombin solution that is mixed at the time of use (Reuthebuch, Lachat et al., 2000; Oz, Rondinone et al., 2003), is superior to Gelfoam-thrombin in cardiac surgeries and has reduced bleeding when used in open nephrectomies and laparoscopy (Guzzo, Pollock et al., 2009). In addition, Kumar, Wickremasinghe et al. (2015) also designed a self-assembled short peptide nanofiber hydrogel SLac (KSLSLRGSLSLS LKGRGDS), which in itself provides a physical barrier to bleeding wounds. Batroxobin is a serine protease derived from snake venom, which can significantly reduce fibrinogen and thrombolysis. SLac loaded with bactretase produced a kind of drug-loaded hydrogel named SB50, which enhanced the role of coagulation. Huri, Dogantekin et al. (2016) mixed ABS hemostatic agent with amphiphilic short peptide (Lauryl -VVAGK-Am) 1:1 to obtain a kind of nano hemostatic agent, which played an excellent hemostatic role in partial nephrectomy.

### Design and fusion of self-assembling peptides

Due to its stable gelatinization, self-assembled short peptide RADA has attracted much attention as a novel hemostatic biomaterial. However, its low pH (3.0–4.0) is easy to cause tissue damage, and the biological activity is unsatisfactory. Because of this, the researchers not only designed various types of self-assembled short peptides, but also fused other peptides or proteins with RADA to obtain new hemostatic biomaterials. It provides methods and ideas for designing and fabricating more hemostatic biomaterials.

RADA16-based fusion peptides have been gradually applied in the field of biomedicine, since RADA16-based fusion peptides have acquired new functions while maintaining the original functions of RADA16. Researchers have designed more excellent RADA16-derived hemostatic biomaterials by selecting different functional sequences from natural active peptides and linking them to RADA16. The short peptide GRGDS mediates cell adhesion by binding to a variety of integrins. The functional sequence YIGSR, which appears in the β1 chain of laminin-1, shows a strong ability for cell adhesion, migration and formation of the endothelial tube (Nomizu, Kim et al., 1995; Chada, Mather et al., 2006). Thus, Cheng, Wu et al. (2013) designed two novel self-assembled short peptides, namely RADA16-GRGDS and RADA16-YIGSR, by linking the functional sequence GRGDS and YIGSR respectively based on the RADA16 self-assembled short peptide.

Most of these functional peptides are small fragment functional motifs of the active protein. Therefore, there are some insurmountable shortcomings (Wang, Wang et al., 2019): 1) With the decrease in the number of functional sequence amino acids, the function of the active protein was greatly reduced. Therefore, it is difficult for small fragment functional motifs to give full play to the function of active proteins. 2) The peptides fused with RADA16 are all chemically synthesized, and the cost of synthesizing and purifying the polypeptide is relatively high. 3) The genetic engineering fermentation is the preferred method for obtaining drug proteins at a low cost. Due to the relatively low molecular weight and poor stability of RADA16 and fusion peptides, it is difficult to obtain the ideal concentration level even if they are over-expressed in host cells by genetic engineering. Therefore, the stability of the expression product is usually improved by fusing RADA-16 with other proteins. Yang, Wei et al. (2018) fused the RADA16 gene to the 3′ end of the open reading frame (ORF) encoding an elastin-like peptide (ELP) by genetic recombination to construct new hemostatic biomaterials: 36ELP-RADA, 60ELP-RADA and 96ELP-RADA. In particular, the 96ELP-RADA sponge film showed a good hemostatic effect. Our group constructed a new hemostatic sponge of HLC-RADA by fusing human-like collagen with RADA16, then lyophilizing the fusion protein, which showed effective hemostasis in a hemostatic model of rabbit liver.

### Flexibility of biomaterials for hemostatic applications

A variety of hemostatic products play an important role in the field of clinical hemostatic application by virtue of their unique advantages. However, the application of these hemostatic biomaterials is often limited by many factors, such as the amount of bleeding, bleeding sites, and different hemostatic effects of the biomaterials, etc. In the face of multiple bleeding conditions, a biomaterial can be made into various forms including granules, solution, powder, hydrogel, film, fiber, and porous sponge, and customized into different product types, such as gauze, spray, and injectable hydrogel. Take chitosan for an example, a high-viscosity chitosan solution can be injected into blood vessels, not only to block blood vessels and prevent blood flow to capillaries, but of little to avoid infection. Chitosan powder can be combined with gauze or made into a spray, which is suitable for emergency treatment with large-area skin damage but small blood loss (Muzzarelli, Morganti et al., 2007). Chitosan fiber not only has good cell compatibility and cell adhesion (Pillai, Paul et al., 2009), but also contributes to the regeneration of skin tissue and the inhibition of scar formation. Chitosan porous materials mainly include porous microspheres, porous fibers, porous sponges, or a combination of the above several types of hemostatic materials (Huang, Sun et al., 2015), the porous structure of which facilitates the discharge of secretion from the wound and tissue healing. Chitosan hydrogel can achieve better and more effective contact with the wound, and provide a moist environment to promote wound healing (Ono, Ishihara et al., 2001). Mi, Shyu et al. (2001) prepared a novel asymmetric chitosan film consisting of a surface with a macroporous sponge-like sub-layer, which can control water loss, has excellent oxygen permeability, and promotes fluid excretion. Meanwhile, due to the presence of a dense cortex and inherent antimicrobial properties of deacetylated chitosan, it can inhibit the invasion of exogenous microorganisms. Furthermore, it has a better hemostatic effect and can accelerate wound healing.

In addition, some researchers have reported that some hemostatic biomaterials could convert from, or strengthen the role of a certain type biomaterials to better facilitate hemostasis, when coming in contact with a bleeding wound. For example, it was reported that CEGP-003 (Bang, Lee et al., 2018) is a mixture of EGF and hydroxyethylcellulose. Considering the Hydroxyethylcellulose with good adhesion and hygroscopicity properties (Lee, Park et al., 2000) could change powder into the adhesive hydrogel, which is beneficial to seal the bleeding site by forming a stable mechanical barrier, it has the potential for the treatment of UGIB (upper gastrointestinal bleeding) and oozing lesions resulted from endoscopic resection. Moreover, a novel matrix hemostatic hydrogel (Hong, Zhou et al., 2019) prepared consisted of methacrylated gelatin (GelMA) and *N*-(2-aminoethyl)-4-(4-(hydroxym ethyl)-2- meth oxy-5-nitrosophenoxy) butanamide (NB) linked to the glycosam inoglycan hyaluronic acid (HA-NB), whose composition is similar to the extracellular matrix (ECM). It not only has the advantage of high absorption of gelatin, but also makes full use of the advantages of HA-NB polymer matrices that has excellent tissue fusion and integration, which help the hydrogel rapid gelling and bond with the tissue surface through UV light irradiation (Yang, Zhang et al., 2016) to seal bleeding sites in arteries and cardiac walls.

## Discussion

Traumas and excessive bleeding are major potential factors for coagulopathy, including persistent hypothermia, metabolic acidosis, and inability to form clots and initiate clotting mechanisms. In this case, the assistance of external force is required to effectively stop bleeding in a short amount of time, so as not to cause other side effects or even life-threatening situations. For this reason, researchers have been studying new hemostatic biomaterials and products to further improve their hemostatic properties in order to meet the hemostatic needs of patients with different bleeding wounds.

In recent years, a variety of hemostatic biological materials and products have been developed, mainly divided into natural biological materials and synthetic biological materials. Some of these biomaterials have only absorptive and passive interactions, while others with active biological interactions promote hemostatic mechanisms. Absorptive and passively interactive biomaterials do not contain any specific components that enhance hemostasis or protect organisms from bacterial infection, but merely serve the purpose of hemostasis by covering wounds and absorbing blood and exudates. For example, oxidized regenerated cellulose (ORC) gauze and starch-based microspheres could seal the wound by absorbing the fluid from the blood to concentrate the effective components (Cheng, He et al., 2016). In this process, the biomaterials improved the hemostasis by promoting the formation of the fibrin clot, which has nothing to do with the clotting cascade. On the other hand, bioactive materials and dressings are systems that adhere to bleeding tissue, primarily through themselves or embedded components to promote hemostasis and prevent infection. It was reported that the negatively charged surface of alginate can induce coagulation initiating *via* the auto-activation of coagulation factor XII (Sperling, Fischer et al., 2009), while the positively charged surface on the chitosan can adhere to platelets *via* charge interaction.

Although all commercial hemostatic products conform to the basic criteria for hemostatic biomaterials, their different hemostatic mechanisms affect the choice and application of hemostatic biomaterials in bleeding patients. In particular, the hemostatic biomaterials that are dependent on the presence of platelet, cannot be applied to bleeding patients with platelet deficiency or dysfunction. In comparison, other hemostatic biomaterials that can activate certain coagulation cascade components independent of platelet presence, are used to stop bleeding in those patients. In addition, different bleeding sites in the body have different requirements for the manner of hemostasis and the form of hemostatic biomaterials. The most common bleeding sites can be divided into compressible hemostasis and non-compressible hemostasis. For it, one of the common methods is to prepare hemostatic biomaterials in different forms, such as spray, powder, solution, hydrogel, etc., making them widely used in hemostasis. When using a hemostatic biomaterial to stop bleeding, the limitations in application should be taken into consideration to avoid side effects. For example, some hemostatic biomaterials cannot be injected intravenously in case they cause vascular embolization.

In addition, hemostatic biomaterials also have a series of problems such as biosafety, hemostatic effect and high cost, which limit their wide application. To prepare hemostatic biomaterials that can meet the needs of patients with hemorrhage, it is necessary to modify the properties of the materials. For example, polysaccharide is natural molecule with advantages such as abundant sources, diversity of size and charge, no immune responses, as well as biodegradability (Swierczewska, Han et al., 2016). Particularly, polysaccharide-based biomaterials that can be easily prepared and modified by chemical or physical methods (Basu, Kunduru et al., 2015). On this basis, the biomaterial can be modified to overcome its original shortcomings and obtain new properties. At present, the commonly used technologies for improving biomaterials include crosslinking technology and gene recombination technology. For instance, the poor mechanical properties of collagen and gelatin can be improved by the cross-linking technology to adapt to hemodynamics and adhere to the tissues. It is vital important to choose a biocompatible, non-toxic cross-linking agent to cross-link the biomaterials in case of inflammation or side effects. On the other hand, the human-like collagen developed through genetic engineering technology not only overcame the disadvantages of the potential risk of the pathogen, and the instabilities between batches, but also possessed good hemostatic performance and the advantages of low cost and large-scale production.

Finally, it is difficult for a single biomaterial to meet all hemostatic requirements in the clinic and the development of hemostatic biomaterials. Because of these difficulties, two main strategies occur. One is to combine two or more hemostatic biomaterials to simultaneously obtain the multifunctional biomaterials. The other is to develop new hemostatic polypeptides or proteins with good biocompatibility and hemostatic effect through gene recombinant technology. It is worth mentioning that self-assembling peptides and their derivatives have attracted more and more attention in the field of hemostasis under their unique advantages. The design and development of self-assembling hemostatic peptides mainly include four aspects: 1) design a peptide matrix scaffold that helps red blood cells aggregation, blood coagulation, and has stronger viscous and compressive capacity. 2) Screen out novel peptides with specific self-assembly properties and hemostasis by the combinatorial chemical library and combined peptide library. 3) Fabricate a multifunctional hemostatic material capable of promoting blood coagulation, cell proliferation, and tissue regeneration by integrating the biological activity sequences such as coagulant procoagulant motifs, cell adhesion motifs and protein-protein interaction motifs into peptides. 4) Blend the synthetic peptides and other hemostatic agents including internal hemostatic drugs, topical hemostatic agents, etc., making them have higher tensile strength, hardness and hemostatic effect. The last but not least, some researchers have focused on developing a hemostatic sealant that has similar components to ECM, and are directed at finding an optimum system that mimics, enhances and even amplifies the clotting mechanism.

## References

[B1] Achneck H. E. Sileshi B. Jamiolkowski R. M. Albala D. M. Shapiro M. L. Lawson J. H. (2010). A comprehensive review of topical hemostatic agents: Efficacy and recommendations for use. Ann. Surg. 251 (2), 217–228. 10.1097/SLA.0b013e3181c3bcca 20010084

[B2] Aydin O. Tuncal S. Kilicoglu B. Onalan A. K. Gonultas M. A. Ozer H. (2015). Effects of Ankaferd Blood Stopper and calcium alginate in experimental model of hepatic parenchymal bleeding. Bratisl. Lek. Listy 116 (2), 128–131. 10.4149/bll_2015_025 25665481

[B3] Bang B. W. Lee D. H. Kim H. K. Kwon K. S. Shin Y. W. Hong S. J. (2018). CEGP-003 spray has a similar hemostatic effect to epinephrine injection in cases of acute upper gastrointestinal bleeding. Dig. Dis. Sci. 63 (11), 3026–3032. 10.1007/s10620-018-5208-z 30054842

[B4] Bano I. Arshad M. Yasin T. Ghauri M. A. Younus M. (2017). Chitosan: A potential biopolymer for wound management. Int. J. Biol. Macromol. 102, 380–383. 10.1016/j.ijbiomac.2017.04.047 28412341

[B5] Basu A. Kunduru K. R. Abtew E. Domb A. J. (2015). Polysaccharide-based conjugates for biomedical applications. Bioconjug Chem. 26 (8), 1396–1412. 10.1021/acs.bioconjchem.5b00242 26106905

[B6] Behrens A. M. Sikorski M. J. Kofinas P. (2014). Hemostatic strategies for traumatic and surgical bleeding. J. Biomed. Mater. Res. Part A 102 (11), 4182–4194. 10.1002/jbm.a.35052 PMC5663245 24307256

[B7] Berrettini M. Mariani G. Schiavoni M. Rocino A. Di Paolantonio T. Longo G. (2001). Pharmacokinetic evaluation of recombinant, activated factor VII in patients with inherited factor VII deficiency. Haematologica 86 (6), 640–645. 11418374

[B8] Bhatia S. K. (2010). Traumatic InjuriesChapter 10Traumatic injuries. New York, NY, USA: Biomaterials for Clinical ApplicationsSpringer, 213–258.

[B9] Blajchman M. A. (2003). Substitutes and alternatives to platelet transfusions in thrombocytopenic patients. J. Thromb. Haemost. 1 (7), 1637–1641. 10.1046/j.1538-7836.2003.00332.x 12871300

[B10] Chada D. Mather T. Nollert M. U. (2006). The synergy site of fibronectin is required for strong interaction with the platelet integrin αIIbβ3. Ann. Biomed. Eng. 34 (10), 1542–1552. 10.1007/s10439-006-9161-1 16933105

[B11] Chan L. W. Kim C. H. Wang X. Pun S. H. White N. J. Kim T. H. (2016). PolySTAT-modified chitosan gauzes for improved hemostasis in external hemorrhage. Acta Biomater. 31, 178–185. 10.1016/j.actbio.2015.11.017 26593785 PMC4728046

[B12] Chandler M. H. Roberts M. Sawyer M. Myers G. (2012). The US military experience with fresh whole blood during the conflicts in Iraq and Afghanistan. Semin. Cardiothorac. Vasc. Anesth. 16 (3), 153–159. 10.1177/1089253212452344 22927704

[B13] Chen C. Zhang Y. Fei R. Cao C. Wang M. Wang J. (2016). Hydrogelation of the short self-assembling peptide I3QGK regulated by transglutaminase and use for rapid hemostasis. ACS Appl. Mater Interfaces 8 (28), 17833–17841. 10.1021/acsami.6b04939 27337106

[B14] Chen Q. Liu Y. Wang T. Wu J. Zhai X. Li Y. (2017). Chitosan–PVA monodisperse millimeter-sized spheres prepared by electrospraying reduce the thromboembolic risk in hemorrhage control. J. Mater. Chem. B 5 (20), 3686–3696. 10.1039/C7TB00032D 32264057

[B15] Cheng T. Y. Wu H. C. Huang M. Y. Chang W. H. Lee C. H. Wang T. W. (2013). Self-assembling functionalized nanopeptides for immediate hemostasis and accelerative liver tissue regeneration. Nanoscale 5 (7), 2734–2744. 10.1039/c3nr33710c 23426280

[B16] Cheng W. He J. Chen M. Li D. Li H. Chen L. (2016). Preparation, functional characterization and hemostatic mechanism discussion for oxidized microcrystalline cellulose and its composites. Fibers Polym. 17 (8), 1277–1286. 10.1007/s12221-016-6279-0

[B17] Csukas D. Urbanics R. Moritz A. Ellis-Behnke R. (2015). AC5 Surgical Hemostat™ as an effective hemostatic agent in an anticoagulated rat liver punch biopsy model. Nanomedicine Nanotechnol. Biol. Med. 11 (8), 2025–2031. 10.1016/j.nano.2015.01.001 25597908

[B18] Dai C. Yuan Y. Liu C. Wei J. Hong H. Li X. (2009). Degradable, antibacterial silver exchanged mesoporous silica spheres for hemorrhage control. Biomaterials 30 (29), 5364–5375. 10.1016/j.biomaterials.2009.06.052 19625081

[B19] de Azevedo C. L. Marques M. M. Bombana A. C. (2003). Cytotoxic effects of cyanoacrylates used as retrograde filling materials: An *in vitro* analysis. Pesqui. Odontol. Bras. 17 (2), 113–118. 10.1590/s1517-74912003000200003 14569351

[B20] Demetri G. D. (2000). Pharmacologic treatment options in patients with thrombocytopenia. Semin. Hematol. 37 (24), 11–18. 10.1016/s0037-1963(00)90048-9 10831284

[B21] Egeli T. Sevinç A. İ. Bora S. Yakut M. C. Cevizci T. Canda T. (2012). Microporous polysaccharide hemospheres and seroma formation after mastectomy and axillary dissection in rats. Balkan Med. J. 29 (2), 179–183. 10.5152/balkanmedj.2012.005 25206991 PMC4115844

[B22] Ellis-Behnke R. G. Liang Y. X. Tay D. K. Kau P. W. Schneider G. E. Zhang S. (2006). Nano hemostat solution: Immediate hemostasis at the nanoscale. Nanomedicine 2 (4), 207–215. 10.1016/j.nano.2006.08.001 17292144

[B23] Ellis-Behnke R. G. Schneider G. E. (2011). Peptide amphiphiles and porous biodegradable scaffolds for tissue regeneration in the brain and spinal cord. Methods Mol. Biol. 726, 259–281. 10.1007/978-1-61779-052-2_17 21424455

[B24] Ersoy G. Kaynak M. F. Yilmaz O. Rodoplu U. Maltepe F. Gokmen N. (2007). Hemostatic effects of microporous polysaccharide hemosphere in a rat model with severe femoral artery bleeding. Adv. Ther. 24 (3), 485–492. 10.1007/bf02848770 17660156

[B25] Etchill E. W. Myers S. P. Raval J. S. Hassoune A. SenGupta A. Neal M. D. (2017). Platelet transfusion in critical care and surgery: Evidence-based review of contemporary practice and future directions. Shock 47 (5), 537–549. 10.1097/shk.0000000000000794 27849676

[B26] Fan D. D. Luo Y. Mi Y. Ma X. X. Shang L. (2005). Characteristics of fed-batch cultures of recombinant *Escherichia coli* containing human-like collagen cDNA at different specific growth rates. Biotechnol. Lett. 27 (12), 865–870. 10.1007/s10529-005-6720-8 16086249

[B27] Farooq F. T. Wong R. C. K. (2005). Injection sclerotherapy for the management of esophageal and gastric varices. Tech. Gastrointest. Endosc. 7 (1), 8–17. 10.1016/j.tgie.2004.10.003

[B28] Fontenot J. D. Rasmussen J. P. Gavin M. A. Rudensky A. Y. (2005). A function for interleukin 2 in Foxp3-expressing regulatory T cells. Nat. Immunol. 6 (11), 1142–1151. 10.1038/ni1263 16227984

[B29] Fouani M. H. Nikkhah M. Mowla J. (2019). Straightforward and cost-effective production of RADA-16I peptide in *Escherichia coli* . Iran. J. Biotechnol. 17 (2), 1–7. 10.21859/ijb.2125 PMC6697845 31457058

[B30] Fries D. Martini W. Z. (2010). Role of fibrinogen in trauma-induced coagulopathy. Br. J. Anaesth. 105 (2), 116–121. 10.1093/bja/aeq161 20627882

[B31] Gao X. R. Xu H. J. Wang L. F. Liu C. B. Yu F. (2017). Mesenchymal stem cell transplantation carried in SVVYGLR modified self-assembling peptide promoted cardiac repair and angiogenesis after myocardial infarction. Biochem. Biophys. Res. Commun. 491 (1), 112–118. 10.1016/j.bbrc.2017.07.056 28709866

[B32] Gorbet M. B. Sefton M. V. (2004). Biomaterial-associated thrombosis: Roles of coagulation factors, complement, platelets and leukocytes. Biomaterials 25 (26), 5681–5703. 10.1016/j.biomaterials.2004.01.023 15147815

[B33] Granville-Chapman J. Jacobs N. Midwinter M. J. (2011). Pre-hospital haemostatic dressings: A systematic review. Injury 42 (5), 447–459. 10.1016/j.injury.2010.09.037 21035118

[B34] Gu B. K. Park S. J. Kim M. S. Lee Y. J. Kim J. I. Kim C. H. (2016). Gelatin blending and sonication of chitosan nanofiber mats produce synergistic effects on hemostatic functions. Int. J. Biol. Macromol. 82, 89–96. 10.1016/j.ijbiomac.2015.10.009 26456289

[B35] Guo H. D. Wang H. J. Tan Y. Z. Wu J. H. (2011). Transplantation of marrow-derived cardiac stem cells carried in fibrin improves cardiac function after myocardial infarction. Tissue Eng. Part A 17 (1-2), 45–58. 10.1089/ten.TEA.2010.0124 20673001

[B36] Guzzo T. J. Pollock R. A. Forney A. Aggarwal P. Matlaga B. R. Allaf M. E. (2009). Safety and efficacy of a surgeon-prepared gelatin hemostatic agent compared with FloSeal for hemostasis in laparoscopic partial nephrectomy. J. Endourol. 23 (2), 279–282. 10.1089/end.2008.0535 19196066

[B37] Harris J. M. (1992). Introduction to biotechnical and biomedical applications of poly(ethylene glycol), Chem. Topics in Applied Chemistry., 8 1–14. 10.1007/978-1-4899-0703-5_1

[B38] Harrison I. P. Spada F. (2018). Hydrogels for atopic dermatitis and wound management: A superior drug delivery vehicle. Pharmaceutics 10 (2), 71. 10.3390/pharmaceutics10020071 29899219 PMC6027388

[B39] Henderson P. W. Kadouch D. J. Singh S. P. Zawaneh P. N. Weiser J. Yazdi S. (2010). A rapidly resorbable hemostatic biomaterial based on dihydroxyacetone. J. Biomed. Mater Res. A 93 (2), 776–782. 10.1002/jbm.a.32586 19653301

[B40] Hickman D. A. Pawlowski C. L. Sekhon U. D. S. Marks J. Gupta A. S. (2018). Biomaterials and advanced technologies for hemostatic management of bleeding. Adv. Mater 30 (4), 1700859. 10.1002/adma.201700859 PMC5831165 29164804

[B41] Hong Y. Zhou F. Hua Y. Zhang X. Ni C. Pan D. (2019). A strongly adhesive hemostatic hydrogel for the repair of arterial and heart bleeds. Nat. Commun. 10 (1), 2060. 10.1038/s41467-019-10004-7 31089131 PMC6517429

[B42] Hu Y. Yu X. Dan W. Yin T. You J. Xiong S. (2017). Preparation, characterization and biomedical applications of collagen based hydrogels. J. Funct. Mater. 5 9733–9743. 10.1021/acsomega.9b04080

[B43] Huang X. Sun Y. Nie J. Lu W. Yang L. Zhang Z. (2015). Using absorbable chitosan hemostatic sponges as a promising surgical dressing. Int. J. Biol. Macromol. 75, 322–329. 10.1016/j.ijbiomac.2015.01.049 25661881

[B44] Humphreys M. R. Castle E. P. Andrews P. E. Gettman M. T. Ereth M. H. (2008). Microporous polysaccharide hemospheres for management of laparoscopic trocar injury to the spleen. Am. J. Surg. 195 (1), 99–103. 10.1016/j.amjsurg.2007.03.006 18070734

[B45] Huri E. Dogantekin E. Hayran M. Malkan U. Y. Ergun M. Firat A. (2016). Ultrastructural analyses of the novel chimeric hemostatic agent generated via nanotechnology, ABS nanohemostat, at the renal tissue level. Springerplus 5 (1), 1931. 10.1186/s40064-016-3625-z 27917335 PMC5101247

[B46] Jiang X. Wang Y. Fan D. Zhu C. Liu L. Duan Z. (2017). A novel human-like collagen hemostatic sponge with uniform morphology, good biodegradability and biocompatibility. J. Biomater. Appl. 31 (8), 1099–1107. 10.1177/0885328216687663 28077050

[B47] Kaufman R. M. Djulbegovic B. Gernsheimer T. Kleinman S. Tinmouth A. T. Capocelli K. E. (2015). Platelet transfusion: A clinical practice guideline from the AABB. Ann. Intern Med. 162 (3), 205–213. 10.7326/m14-1589 25383671

[B48] Kauvar D. S. Holcomb J. B. Norris G. C. Hess J. R. (2006). Fresh whole blood transfusion: A controversial military practice. J. Trauma 61 (1), 181–184. 10.1097/01.ta.0000222671.84335.64 16832268

[B49] Kheirabadi B. (2011). Evaluation of topical hemostatic agents for combat wound treatment. US Army Med Dep J 2, 25–37. 21607904

[B50] Komatsu S. Nagai Y. Naruse K. Kimata Y. (2014). The neutral self-assembling peptide hydrogel SPG-178 as a topical hemostatic agent. PLoS One 9 (7), e102778. 10.1371/journal.pone.0102778 25047639 PMC4105593

[B51] Kommu S. S. McArthur R. Emara A. M. Reddy U. D. Anderson C. J. Barber N. J. (2015). Current status of hemostatic agents and sealants in urologic surgical practice. Rev. urology 17 (3), 150–159. 10.3909/riu0673 PMC4633658 26543429

[B52] Kondo Y. Nagasaka T. Kobayashi S. Kobayashi N. Fujiwara T. (2014). Management of peritoneal effusion by sealing with a self-assembling nanofiber polypeptide following pelvic surgery. Hepatogastroenterology 61 (130), 349–353. 10.5754/hge121000 24901138

[B53] Kumar V. A. Taylor N. L. Jalan A. A. Hwang L. K. Wang B. K. Hartgerink J. D. (2014). A nanostructured synthetic collagen mimic for hemostasis. Biomacromolecules 15 (4), 1484–1490. 10.1021/bm500091e 24694012 PMC3993945

[B54] Kumar V. A. Wickremasinghe N. C. Shi S. Hartgerink J. D. (2015). Nanofibrous snake venom hemostat. ACS Biomater. Sci. Eng. 1 (12), 1300–1305. 10.1021/acsbiomaterials.5b00356 26753175 PMC4704453

[B55] Lan G. Lu B. Wang T. Wang L. Chen J. Yu K. (2015). Chitosan/gelatin composite sponge is an absorbable surgical hemostatic agent. Colloids Surfaces B Biointerfaces 136, 1026–1034. 10.1016/j.colsurfb.2015.10.039 26590895

[B56] Laurenti J. B. Zazeri G. Povinelli A. P. R. de Godoy M. F. Braile D. M. da Rocha T. ó. R. F. (2017). Enhanced pro-coagulant hemostatic agents based on nanometric zeolites. Microporous Mesoporous Mater. 239, 263–271. 10.1016/j.micromeso.2016.10.020

[B57] Lee J. W. Park J. H. Robinson J. R. (2000)., 89. 2: CO, 850–866. 10.1002/1520-6017(200007)89:7<850:AID-JPS2>3.0 Bioadhesive‐based dosage forms: The next generation, J. Pharm. Sci. 10861586

[B58] Levy J. H. Goodnough L. T. (2015). How I use fibrinogen replacement therapy in acquired bleeding. Blood 125 (9), 1387–1393. 10.1182/blood-2014-08-552000 25519751

[B59] Lewis K. M. Kuntze C. E. Gulle H. (2015). Control of bleeding in surgical procedures: Critical appraisal of HEMOPATCH (sealing hemostat). N.Z.) 9, 1–10. 10.2147/MDER.S90591 PMC4694675 26730213

[B60] Li G. Quan K. Liang Y. Li T. Yuan Q. Tao L. (2016). Graphene-montmorillonite composite sponge for safe and effective hemostasis. ACS Appl. Mater Interfaces 8 (51), 35071–35080. 10.1021/acsami.6b13302 27935296

[B61] Li J. Cao W. Lv X. X. Jiang L. Li Y. J. Li W. Z. (2013). Zeolite-based hemostat QuikClot releases calcium into blood and promotes blood coagulation *in vitro* . Acta Pharmacol. Sin. 34 (3), 367–372. 10.1038/aps.2012.159 23334236 PMC4002488

[B62] Liang Y. Xu C. Li G. Liu T. Liang J. F. Wang X. (2018). Graphene-kaolin composite sponge for rapid and riskless hemostasis. Colloids Surf. B Biointerfaces 169, 168–175. 10.1016/j.colsurfb.2018.05.016 29763772

[B63] Lumsden A. B. Heyman E. R. (2006). Prospective randomized study evaluating an absorbable cyanoacrylate for use in vascular reconstructions. J. Vasc. Surg. 44 (5), 1002–1009.e1. 10.1016/j.jvs.2006.06.039 17020801

[B64] Luo Z. Wang S. Zhang S. (2011). Fabrication of self-assembling D-form peptide nanofiber scaffold d-EAK16 for rapid hemostasis. Biomaterials 32 (8), 2013–2020. 10.1016/j.biomaterials.2010.11.049 21167593

[B65] Masci E. Faillace G. Longoni M. (2018). Use of oxidized regenerated cellulose to achieve hemostasis during laparoscopic cholecystectomy: A retrospective cohort analysis. BMC Res. Notes 11 (1), 239. 10.1186/s13104-018-3344-3 29642951 PMC5896066

[B66] Masuhara H. Fujii T. Watanabe Y. Koyama N. Tokuhiro K. (2012). Novel infectious agent-free hemostatic material (TDM-621) in cardiovascular surgery. Ann. Thorac. Cardiovasc Surg. 18 (5), 444–451. 10.5761/atcs.oa.12.01977 22986759

[B67] Mi F. L. Shyu S. S. Wu Y. B. Lee S. T. Shyong J. Y. Huang R. N. (2001). Fabrication and characterization of a sponge-like asymmetric chitosan membrane as a wound dressing. Biomaterials 22 (2), 165–173. 10.1016/s0142-9612(00)00167-8 11101160

[B68] Mie M. Oomuro M. Kobatake E. (2013). Hydrogel scaffolds composed of genetically synthesized self-assembling peptides for three-dimensional cell culture. Polym. J. 45 (5), 504–508. 10.1038/pj.2012.216

[B69] Montanaro L. Arciola C. R. Cenni E. Ciapetti G. Savioli F. Filippini F. (2000). Cytotoxicity, blood compatibility and antimicrobial activity of two cyanoacrylate glues for surgical use. Biomaterials 22 (1), 59–66. 10.1016/S0142-9612(00)00163-0 11085384

[B70] Motlagh D. Yang J. Lui K. Y. Webb A. R. Ameer G. A. (2006). Hemocompatibility evaluation of poly(glycerol-sebacate) *in vitro* for vascular tissue engineering. Biomaterials 27 (24), 4315–4324. 10.1016/j.biomaterials.2006.04.010 16675010

[B71] Muzzarelli R. A. A. Morganti P. Morganti G. Palombo P. Palombo M. Biagini G. (2007). Chitin nanofibrils/chitosan glycolate composites as wound medicaments. Carbohydr. Polym. 70 (3), 274–284. 10.1016/j.carbpol.2007.04.008

[B72] Nagamatsu M. Podratz J. Windebank A. J. Low P. A. (1997). Acidity is involved in the development of neuropathy caused by oxidized cellulose. J. Neurol. Sci. 146 (2), 97–102. 10.1016/s0022-510x(96)00295-x 9077504

[B73] Nomizu M. Kim W. H. Yamamura K. Utani A. Song S. Y. Otaka A. (1995). Identification of cell binding sites in the laminin alpha 1 chain carboxyl-terminal globular domain by systematic screening of synthetic peptides. J. Biol. Chem. 270 (35), 20583–20590. 10.1074/jbc.270.35.20583 7657636

[B74] Ono K. Ishihara M. Ozeki Y. Deguchi H. Sato M. Saito Y. (2001). Experimental evaluation of photocrosslinkable chitosan as a biologic adhesive with surgical applications. Surgery 130 (5), 844–850. 10.1067/msy.2001.117197 11685194

[B75] Ostomel T. A. Shi Q. Tsung C. K. Liang H. Stucky G. D. (2006). Spherical bioactive glass with enhanced rates of hydroxyapatite deposition and hemostatic activity. Small 2 (11), 1261–1265. 10.1002/smll.200600177 17192971

[B76] Overby R. J. Feldman D. S. (2018). Influence of poly(ethylene glycol) end groups on poly(ethylene glycol)-albumin system properties as a potential degradable tissue scaffold. J. Funct. Biomater. 10 (1), 1. 10.3390/jfb10010001 30586909 PMC6462978

[B77] Oz M. C. Rondinone J. F. Shargill N. S. (2003). FloSeal matrix: New generation topical hemostatic sealant. J. Card. Surg. 18 (6), 486–493. 10.1046/j.0886-0440.2003.00302.x 14992097

[B78] Pan H. Fan D. Cao W. Zhu C. Duan Z. Fu R. (2017). Preparation and characterization of breathable hemostatic hydrogel dressings and determination of their effects on full-thickness defects. Polymers 9 (12), 727. 10.3390/polym9120727 30966027 PMC6418977

[B79] Pereira B. M. Bortoto J. B. Fraga G. P. (2018)., 45. Rev. do Colégio Bras. Cir. Agentes hemostáticos tópicos em cirurgia: Revisão e perspectivas, 10.1590/0100-6991e-20181900 30365692

[B80] Pillai C. K. S. Paul W. Sharma C. P. (2009). Chitin and chitosan polymers: Chemistry, solubility and fiber formation. Prog. Polym. Sci. 34 (7), 641–678. 10.1016/j.progpolymsci.2009.04.001

[B81] Piozzi G. N. Reitano E. Panizzo V. Rubino B. Bona D. Tringali D. (2018). Practical suggestions for prevention of complications arising from oxidized cellulose retention: A case report and review of the literature. Am. J. Case Rep. 19, 812–819. 10.12659/ajcr.910060 29991675 PMC6066980

[B82] Pourshahrestani S. Zeimaran E. Djordjevic I. Kadri N. A. Towler M. R. (2016). Inorganic hemostats: The state-of-the-art and recent advances. Mater Sci. Eng. C Mater Biol. Appl. 58, 1255–1268. 10.1016/j.msec.2015.09.008 26478429

[B83] Pourshahrestani S. Zeimaran E. Kadri N. A. Gargiulo N. Jindal H. M. Naveen S. V. (2017). Potency and cytotoxicity of a novel gallium-containing mesoporous bioactive glass/chitosan composite scaffold as hemostatic agents. ACS Appl. Mater Interfaces 9 (37), 31381–31392. 10.1021/acsami.7b07769 28836753

[B84] Pusateri A. E. McCarthy S. J. Gregory K. W. Harris R. A. Cardenas L. McManus A. T. (2003). Effect of a chitosan-based hemostatic dressing on blood loss and survival in a model of severe venous hemorrhage and hepatic injury in swine. J. Trauma 54 (1), 177–182. 10.1097/00005373-200301000-00023 12544915

[B85] Rad-Malekshahi M. Lempsink L. Amidi M. Hennink W. E. Mastrobattista E. (2016). Biomedical applications of self-assembling peptides. Bioconjug Chem. 27 (1), 3–18. 10.1021/acs.bioconjchem.5b00487 26473310

[B86] Rahe-Meyer N. Sorensen B. (2011). For: Fibrinogen concentrate for management of bleeding. J. Thromb. Haemost. 9 (1), 1–5. 10.1111/j.1538-7836.2010.04099.x 20946151

[B87] Reuthebuch O. Lachat M. L. Vogt P. Schurr U. Turina M. (2000). FloSeal®: Ein neuartiges Hämostyptikum in der peripheren Gefäßchirurgie. Vasa 29 (3), 204–206. 10.1024/0301-1526.29.3.204 11037719

[B88] Roberts H. R. (2001). Recombinant factor VIIa (Novoseven) and the safety of treatment. Semin. Hematol. 38 (412), 48–50. 10.1016/s0037-1963(01)90148-9 11735111

[B89] Ruan L. Zhang H. Luo H. Liu J. Tang F. Shi Y. K. (2009). Designed amphiphilic peptide forms stable nanoweb, slowly releases encapsulated hydrophobic drug, and accelerates animal hemostasis. Proc. Natl. Acad. Sci. U. S. A. 106 (13), 5105–5110. 10.1073/pnas.0900026106 19289834 PMC2663994

[B90] Salinas A. J. Shruti S. Malavasi G. Menabue L. Vallet-Reg├¾ M. (2011). Substitutions of cerium, gallium and zinc in ordered mesoporous bioactive glasses. Acta Biomater. 7 (9), 3452–3458. 10.1016/j.actbio.2011.05.033 21672640

[B91] Seon G. M. Lee M. H. Kwon B.-J. Kim M. S. Koo M.-A. Seomun Y. (2018). Recombinant batroxobin-coated nonwoven chitosan as hemostatic dressing for initial hemorrhage control. Int. J. Biol. Macromol. 113, 757–763. 10.1016/j.ijbiomac.2018.03.017 29514041

[B92] Seyednejad H. Imani M. Jamieson T. Seifalian A. M. (2008). Topical haemostatic agents. Br. J. Surg. 95 (10), 1197–1225. 10.1002/bjs.6357 18763249

[B93] Shahverdi S. Hajimiri M. Esfandiari M. A. Larijani B. Atyabi F. Rajabiani A. (2014). Fabrication and structure analysis of poly(lactide-co-glycolic acid)/silk fibroin hybrid scaffold for wound dressing applications. Int. J. Pharm. 473 (1), 345–355. 10.1016/j.ijpharm.2014.07.021 25051110

[B94] Shruti S. Salinas A. J. Ferrari E. Malavasi G. Lusvardi G. Doadrio A. L. (2013). Curcumin release from cerium, gallium and zinc containing mesoporous bioactive glasses. Microporous Mesoporous Mater. 180, 92–101. 10.1016/j.micromeso.2013.06.014

[B95] Shruti S. Salinas A. J. Lusvardi G. Malavasi G. Menabue L. Vallet-Regi M. (2013). Mesoporous bioactive scaffolds prepared with cerium-gallium- and zinc-containing glasses. Acta Biomater. 9 (1), 4836–4844. 10.1016/j.actbio.2012.09.024 23026489

[B96] Skopinska-Wisniewska J. Sionkowska A. Kaminska A. Kaznica A. Jachimiak R. Drewa T. (2009). Surface characterization of collagen/elastin based biomaterials for tissue regeneration. Appl. Surf. Sci. 255 (19), 8286–8292. 10.1016/j.apsusc.2009.05.127

[B97] Sperling C. Fischer M. Maitz M. F. Werner C. (2009). Blood coagulation on biomaterials requires the combination of distinct activation processes. Biomaterials 30 (27), 4447–4456. 10.1016/j.biomaterials.2009.05.044 19535136

[B98] Spinella P. C. Perkins J. G. Grathwohl K. W. Repine T. Beekley A. C. Sebesta J. (2007). Risks associated with fresh whole blood and red blood cell transfusions in a combat support hospital. Crit. Care Med. 35 (11), 2576–2581. 10.1097/01.Ccm.0000285996.65226.A9 17828033

[B99] Spitalnik S. L. Triulzi D. Devine D. V. Dzik W. H. Eder A. F. Gernsheimer T. (2015). 2015 proceedings of the national heart, lung, and blood institute's state of the science in transfusion medicine symposium. Transfusion 55 (9), 2282–2290. 10.1111/trf.13250 26260861 PMC4573332

[B100] Sun X. Tang Z. Pan M. Wang Z. Yang H. Liu H. (2017). Chitosan/kaolin composite porous microspheres with high hemostatic efficacy. Carbohydr. Polym. 177, 135–143. 10.1016/j.carbpol.2017.08.131 28962752

[B101] Swierczewska M. Han H. S. Kim K. Park J. H. Lee S. (2016). Polysaccharide-based nanoparticles for theranostic nanomedicine. Adv. Drug Deliv. Rev. 99 (Pt A), 70–84. 10.1016/j.addr.2015.11.015 26639578 PMC4798864

[B102] Tam T. Harkins G. Dykes T. Gockley A. Davies M. (2014). Oxidized regenerated cellulose resembling vaginal cuff abscess. Jsls 18 (2), 353–356. 10.4293/108680813x13693422518597 24960506 PMC4035653

[B103] Thongrong C. Kasemsiri P. Carrau R. L. Bergese S. D. (2013). Control of bleeding in endoscopic skull base surgery: Current concepts to improve hemostasis. ISRN Surg. 2013, 1–11. 10.1155/2013/191543 PMC3697291 23844295

[B104] Valeri C. R. (2006). Letters to the editor. J. Trauma 61 (1), 240–241. 10.1097/01.ta.0000224109.60301.a8 16832287

[B105] Verissimo D. M. Leitao R. F. Ribeiro R. A. Figueiro S. D. Sombra A. S. Goes J. C. (2010). Polyanionic collagen membranes for guided tissue regeneration: Effect of progressive glutaraldehyde cross-linking on biocompatibility and degradation. Acta Biomater. 6 (10), 4011–4018. 10.1016/j.actbio.2010.04.012 20433958

[B106] Wang R. Wang Z. Guo Y. Li H. Chen Z. (2019). Design of a RADA16-based self-assembling peptide nanofiber scaffold for biomedical applications. J. Biomater. Sci. Polym. Ed. 30 (9), 713–736. 10.1080/09205063.2019.1605868 31018781

[B107] Wang T. Zhong X. Wang S. Lv F. Zhao X. (2012). Molecular mechanisms of RADA16-1 peptide on fast stop bleeding in rat models. Int. J. Mol. Sci. 13 (11), 15279–15290. 10.3390/ijms131115279 23203125 PMC3509641

[B108] Watrowski R. Jager C. Forster J. (2017). Improvement of perioperative outcomes in major gynecological and gynecologic-oncological surgery with hemostatic gelatin-thrombin matrix. Vivo 31 (2), 251–258. 10.21873/invivo.11053 PMC5411753 28358708

[B109] Xie Y. Yi Z. X. Wang J. X. Hou T. G. Jiang Q. (2018). Carboxymethyl konjac glucomannan - crosslinked chitosan sponges for wound dressing. Int. J. Biol. Macromol. 112, 1225–1233. 10.1016/j.ijbiomac.2018.02.075 29454058

[B110] Yamawaki I. Taguchi Y. Komasa S. Tanaka A. Umeda M. (2017). Effects of glucose concentration on osteogenic differentiation of type II diabetes mellitus rat bone marrow-derived mesenchymal stromal cells on a nano-scale modified titanium. J. Periodontal Res. 52 (4), 761–771. 10.1111/jre.12446 28321876

[B111] Yan T. Cheng F. Wei X. Huang Y. He J. (2017). Biodegradable collagen sponge reinforced with chitosan/calcium pyrophosphate nanoflowers for rapid hemostasis. Carbohydr. Polym. 170, 271–280. 10.1016/j.carbpol.2017.04.080 28521997

[B112] Yang S. Wei S. Mao Y. Zheng H. Feng J. Cui J. (2018). Novel hemostatic biomolecules based on elastin-like polypeptides and the self-assembling peptide RADA-16. BMC Biotechnol. 18 (1), 12. 10.1186/s12896-018-0422-5 29514614 PMC5842521

[B113] Yang X. Liu W. Li N. Wang M. Liang B. Ullah I. (2017). Design and development of polysaccharide hemostatic materials and their hemostatic mechanism. Biomaterials Sci. 5 (12), 2357–2368. 10.1039/C7BM00554G 29019480

[B114] Yang Y. Zhang J. Liu Z. Lin Q. Liu X. Bao C. (2016). Tissue-integratable and biocompatible photogelation by the imine crosslinking reaction. Adv. Mater 28 (14), 2724–2730. 10.1002/adma.201505336 26840751

[B115] Yu L. Shang X. Chen H. Xiao L. Zhu Y. Fan J. (2019). A tightly-bonded and flexible mesoporous zeolite-cotton hybrid hemostat. Nat. Commun. 10 (1), 1932. 10.1038/s41467-019-09849-9 31036816 PMC6488602

[B116] Yuan H. Chen L. Hong F. F. (2020). A biodegradable antibacterial nanocomposite based on oxidized bacterial nanocellulose for rapid hemostasis and wound healing. ACS Appl. Mater. Interfaces 12 (3), 3382–3392. 10.1021/acsami.9b17732 31880915

